# When formal management practices meet informal governance: family ownership and productivity in UK firms

**DOI:** 10.1007/s11187-026-01191-x

**Published:** 2026-04-14

**Authors:** Yuchen Feng, Andrew Henley, Anna Kochanova

**Affiliations:** Cardiff Business School, Cardiff University, Cardiff, UK

**Keywords:** Structured management practices, Labour productivity, Family ownership, The Management and Expectation Survey, D22, L25, M11

## Abstract

This study examines whether family governance moderates the productivity returns to structured management practices. Using combined data from the UK Management and Expectations Survey (MES) and the Annual Respondents Database X (ARDx) and applying a reformulated Mundlak model, we show that structured management practices are positively associated with labour productivity, but that family ownership significantly weakens their long-run productivity returns. This negative moderating effect is stronger for incentives-related and target-setting practices and is more pronounced among small and medium-sized enterprises and service-sector firms. Overall, our findings highlight execution credibility as a central mechanism linking firm governance structures to the economic returns of formal management systems.

## Abstract (plain language summary)

Formal management practices such as targets, key performance indicators (KPIs), and incentive schemes are widely promoted as tools to boost productivity, yet our study shows that they do not deliver the same long-run benefits across firms. Using UK firm-level data, we find that while structured management practices are generally associated with higher productivity, their long-run benefits are significantly weaker in family-owned firms, because informal family governance can undermine consistent and credible execution over time. This effect is especially pronounced for practices that rely on impartial performance evaluation and rule-based enforcement, as well as among SMEs and service-sector firms. The main implication of this study is that improving productivity requires not only the adoption of better management practices but also strengthening governance structures that support their effective execution, particularly in family firms.

## Introduction

Existing studies widely regard structured management practices as a key input in production function that enhances firm productivity (Bloom & Van Reenen, 2007; Bloom et al., 2019; Broszeit et al., 2019). Formal practices such as target setting, performance monitoring, and incentive systems help reduce internal information asymmetries, improve worker effort, and raise productive efficiency (Gosnell et al., 2020). Compared with physical capital or technological investments, structured management practices are relatively easy to replicate and measure (Scur et al., 2024) and are therefore considered an important institutional source of productivity differences across firms.

Despite their positive average effects, the productivity returns to management practices vary substantially across firms. This heterogeneity suggests that the effectiveness of management practices depends not only on their formal design, but also on the organisational environments in which they are embedded. Management practices require stable, consistent, and predictable execution to generate sustained productivity gains. When formal systems operate in execution environments characterised by discretionary intervention or selective enforcement, their potential productivity benefits are unlikely to be fully realised (Prado, 2011).

Family firms provide a typical context for examining such execution environments. They are characterised by distinctive organisational arrangements in which informal governance, relational control, and concentrated authority coexist with formal management systems (Mustakallio et al., 2002). On the one hand, family firms are often associated with lower agency costs, stronger long-term orientation, and high organisational commitment (Chrisman et al., 2017). On the other hand, a large literature documents limited management professionalisation, flexible interpretation of formal rules, and selective enforcement of procedures in family firms (Owusu-Acheampong et al., 2025; Stewart & Hitt, 2012). As a result, even when structured management practices are formally adopted, their execution may be shaped by informal governance and discretionary intervention.

In this paper, we build on a theory of property rights of family firms (Chrisman et al., 2025) and the role of residual control rights under incomplete contracting, and examine how family ownership conditions the relationship between structured management practices and labour productivity. To this end we link establishment-level data on management practices from the UK Management and Expectations Survey (MES) with labour productivity data from the Annual Respondents Database (ARDx).^1^ We then employ a reformulated Mundlak model (Bell et al., 2019; Mundlak, 1978) that allows for the separate estimation of short-term (within-firm) and long-term (between-firm) effects of management quality.

Our results show that structured management practices are positively associated with labour productivity on average. However, family ownership significantly weakens the long-term productivity returns (between-firm effects) to management practices, while leaving short-term effects unaffected. This suggests that family firms can benefit from management improvements in the short run, but face constraints in transforming these practices into stable and durable organisational capabilities. Notably, this moderating effect persists regardless of whether firms are managed by family members or non-family professionals, and it is more pronounced for incentive-based and target-setting practices. The effect is also stronger among small and medium-sized enterprises (SMEs) and in service-sector firms, where governance infrastructure is typically weaker and performance outcomes are harder to verify.

This paper contributes to the literature in two important ways. First, it advances the management practices and productivity literature by reframing management practices as an execution-dependent organisational capital. While existing studies find heterogeneity in the management–productivity relationship (Bloom et al., 2012b; Broszeit et al., 2019), they offer limited insight into the organisational conditions under which management practices can be credibly executed. By shifting the focus from formal adoption to execution credibility, this study provides new empirical evidence on why similar management practices generate different long-run productivity outcomes across firms. In addition, using linked data from the UK MES and ARDx, we offer a comprehensive evidence for a developed-country context. We also extend the analysis to service-sector firms, which have received less attention in the existing literature.^2^

Second, building on the property rights theory of the family firm (see Chrisman et al. (2025) for a comprehensive review), this paper contributes to the governance–management literature by highlighting the role of residual control rights under incomplete contracting in shaping the effectiveness of formal management systems. When formal rules cannot specify all contingencies, their effectiveness depends critically on who retains the authority to interpret, override, and enforce them ex post. Family ownership, by concentrating residual control rights and expanding the scope for discretionary intervention, may weaken the execution credibility of formal management systems. While prior research on family firms has emphasised informal governance, relational control, and the role of professional managers (Aguilera et al., 2025; Scholes & Wilson, 2014), empirical evidence on how family governance affects the functioning of formal management systems remains limited. We provide new evidence consistent with a mechanism in which discretionary ex post intervention, enabled by concentrated residual control rights, is associated with lower execution credibility of structured management practices and, in turn, weaker long-run productivity returns.

This paper is organised as follows. Section 2 presents the theoretical framework and hypotheses. Section 3 describes the empirical strategy and data. Section 4 reports the main results and robustness checks. Section 5 concludes with policy implications, limitations, and directions for future research.

## Theoretical framework

### Structural management practices as execution-dependent organisational capital

Structured management practices constitute a form of replicable and scalable organisational capital (Attig & Cleary, 2014; Bloom et al., 2019). By establishing explicit goal setting, systematic performance monitoring, formal incentive schemes, and continuous employee training, firms can improve internal coordination, and more effectively translate individual effort into productivity gains (Ichniowski & Shaw, 2003; Koch & McGrath, 1996).

Unlike tangible capital inputs, management practices do not generate immediate economic benefits simply through formal implementation (Bloom et al., 2016; Brynjolfsson et al., 2002). Rather, they are embedded in regular operations, and their economic value depends critically on repeated execution and organisational internalisation over time (Blader et al., 2019). Structured management practices may therefore affect firm productivity through several interrelated channels. First, targets and performance measures improve information flows and align actions across organisational levels (Bellisario et al., 2021). Second, monitoring and incentives strengthen effort provision and reduce shirking and misallocation (Bradbury, 2019). Third, training and standardised routines facilitate organisational learning and diffusion of effective practices (Aragón et al., 2014). Through these channels, structured management practices are expected to improve firm productivity.

However, the effectiveness of management practices hinges on the organisation’s ability to sustain credible commitments in execution (Paşaoğlu, 2015). This requires credible commitments in implementation: targets and evaluations must be applied consistently over time, and incentive schemes must be perceived as fair and rule-based rather than subject to arbitrary revision (Christ, 2013).

These considerations imply that productivity effects of structured management practices do not stem solely from institutional design but depend on how execution authority and managerial discretion are exercised within the firm. In this sense, management practices constitute an execution-dependent form of organisational capital, whose economic value is sensitive to the stability and credibility of internal enforcement. Where firms can sustain stable execution and credible commitments over time, structured management practices are expected to be positively associated with labour productivity. Based on this reasoning, we propose the following hypothesis:

#### Hypothesis 1 (H1)

Structured management practices are positively associated with labour productivity.

### The role of family ownership

Once structured management practices are understood as an execution-dependent form of organisational capital, a central question is whether formal management systems can sustain credible commitments in execution under different ownership structures.

In the general management literature, agency and stewardship theories offer important insights into how governance arrangements shape managerial incentives and behaviour (Davis et al., 1997; Jensen & Meckling, 1979). However, these perspectives primarily focus on motivational alignment and control at the level of owners, top managers, or boards. As such, they offer limited guidance on how governance structures affect the execution of firm-wide formal management systems once these systems are in place.

We build on a property rights theory under incomplete contracting, which emphasises residual control rights as the authority to make decisions and grant exceptions in contingencies not fully specified by formal agreements (Aghion & Holden, 2011; Aguilera et al., 2025). The property rights theory of the family firm (Chrisman et al., 2025) highlights how residual control rights under incomplete contracting shape the credibility of rule-based organisational arrangements. We apply this logic to the execution of firm-wide management systems and argue that the allocation and exercise of residual control rights define the credibility with which formal rules are enforced ex post. From this perspective, the productivity value of structured management practices depends on whether organisational members expect performance criteria, rewards, and sanctions to be applied consistently, rather than adjusted through discretionary intervention.

In family firms, ownership, control, and managerial authority are often highly overlapping, with family owners retaining substantial residual control rights alongside formal decision rights (Mustakallio et al., 2002). Residual control rights are more likely to be exercised directly by controlling family members, increasing the scope for discretionary ex post intervention in the execution of formal management systems (Helsen et al., 2017; Martinez Ferrero et al., 2016). Family controllers may reinterpret performance criteria, override incentive outcomes, or grant exceptions after outcomes are realised. Repeated instances of selective enforcement and informal adjustment may provide organisational members with clear signals. Because of such discretionary interventions, they can rationally discount the binding force of formal commitments, weakening the behavioural effectiveness of targets, incentives, and promotion criteria (Lubatkin et al., 2007).

This distortion of execution credibility is further reinforced by two structural features commonly associated with family ownership, both of which shape the incentives and feasibility of exercising residual control rights under incomplete contracting. First, family firms typically pursue multiple objectives beyond short-term economic performance, including control stability, intergenerational succession, family cohesion, and reputation preservation (Diaz-Moriana et al., 2024; Kotlar & De Massis, 2013) When formal performance constraints embedded in management systems conflict with these non-economic objectives, family owners face stronger incentives to exercise residual control rights selectively (De Massis et al., 2018).

Second, family ownership is associated with a greater reliance on relational governance alongside formal contractual arrangements (Mustakallio et al., 2002). While relational governance can foster trust and long-term cooperation (Steier, 2001), it also lowers the costs of discretionary execution. When internal order is shaped by identity, loyalty, and long-standing relationships, formal performance evaluation and reward allocation are more easily adjusted to accommodate relational considerations. This flexibility reduces the transparency and consistency of rule-based enforcement, further weakening the execution credibility of formal management systems (Zellweger et al., 2019).

Taken together, family ownership does not eliminate the potential value of structured management practices. Formal management systems may still improve coordination, information flows, and organisational learning in family firms. However, the concentration of residual control rights, combined with stronger incentives and opportunities for discretionary execution, may systematically reduce the efficiency with which formal management practices translate into productivity returns. Accordingly, we propose the following moderating hypothesis:

#### Hypothesis 2 (H2)

Relative to non-family firms, family ownership weakens the productivity returns to structured management practices.

### Heterogeneity in the moderating effect of family ownership

Family firms are widely recognised as highly heterogeneous in their governance structures, and organisational arrangements, implying that the mechanisms through which family ownership shapes organisational outcomes are unlikely to be uniform across contexts (Daspit et al., 2021). Residual control rights are therefore central to our analysis, as they grant ultimate authority over resource allocation and institutional enforcement. Their economic relevance depends on how strongly a given management practice relies on credible execution and on whether it meaningfully constrains the discretion of actors closely connected to the holder of residual control rights.

Management practices differ in their dependence on governance structures that sustain credible execution (Fiss, 2008). One class of practices centres on performance commitments and distributive allocation rules, including quantified target setting, performance appraisal, incentive pay, and promotion criteria. The effectiveness of these practices rests on two critical conditions. First, performance outcomes must be sufficiently verifiable to support ex post evaluation (Verbeeten, 2008). Second, rewards and sanctions must be applied consistently over time so that expectations regarding effort–return mappings remain stable (Favero et al., 2014). When residual control rights permit ex post reinterpretation, selective enforcement, or discretionary reallocation of rewards, these conditions become fragile (Hart & Moore, 2007). Anticipating discretionary exceptions, organisational members discount the credibility of formal incentives, weakening the disciplinary role of targets and performance-based rewards.

By contrast, monitoring, feedback, and training practices rely less directly on distributive enforcement and formal commitment. Their primary function is not to allocate rents or impose sanctions, but to improve information flows, reduce process deviations, and facilitate gradual capability accumulation (Schnieder et al., 2025). Because these practices generate efficiency gains through repetition, learning-by-doing, and incremental coordination improvements rather than through contingent reward allocation, their effectiveness is less vulnerable to ex post renegotiation (Hoffer Gittell, 2002). Even in governance environments characterised by relational discretion, such practices can retain productivity benefits without requiring strong commitment to fixed incentive rules. Therefore, our next hypothesis is:

#### Hypothesis 3a (H3a)

The negative moderating effect of family ownership is stronger for management practices that rely on formal performance targets and incentive commitments than for practices related to monitoring and training.

Execution discretion also exerts heterogeneous effects depending on which organisational actors are targeted by management practices. Practices primarily directed at managers, such as managerial performance evaluation, target cascading, and promotion-linked incentives, are designed to constrain decision authority and align managerial discretion with organisational objectives. Their effectiveness therefore depends critically on governance mechanisms that credibly limit managerial autonomy. In family firms, managerial authority frequently overlaps with family control or remains strongly shaped by family preferences and informal authority structures (Bennedsen et al., 2010). As a result, when formal management systems seek to discipline managerial behaviour, the scope for execution distortion is particularly pronounced, as these systems directly impinge upon the core domain of residual control rights (James, 1999).

By contrast, management practices targeting non-managerial employees are less directly connected to the allocation and exercise of residual control rights. While execution discretion may still generate efficiency losses at the operational level, it is less likely to involve systematic reinterpretation of institutional intent or authority boundaries. Consequently, the effectiveness of employee-targeted practices should be less sensitive to governance characteristics associated with family ownership. This study then tests the following hypotheses:

#### Hypothesis 3b (H3b)

The negative moderating effect of family ownership is stronger for management practices targeting managers than for those targeting non-managerial employees.

The property rights framework also suggests that the strength of execution discretion may vary systematically across organisational and technological contexts. In SMEs, formal governance structures are typically weaker (Kussudyarsana et al., 2019). Limited separation between ownership and management, together with less institutionalised HR and compliance functions, reduces the visibility and accountability of deviations from formal rules (Innes & Wiesner, 2012). As a result, family controllers can more easily exercise residual control rights ex post through discretionary interpretation or selective enforcement of formal rules. This weakens execution credibility and constrains the productivity returns to structured management practices. By contrast, in larger firms, more formalised governance arrangements and professional management structures increase the cost of discretionary intervention, attenuating the moderating effect of family ownership (Fang et al., 2016).

Industry characteristics may further condition the operation of residual control rights by shaping the degree of execution discretion embedded in production processes. In manufacturing sector, more standardised processes and more measurable outputs allow formal practices to be integrated into routines and technologies, limiting the room for discretionary reinterpretation at the execution stage (Rao, 2013). In contrast, service sectors rely more heavily on subjective judgement, interpersonal interaction, and difficult-to-measure outputs (Millar & Choi, 2011). The resulting ambiguity in performance evaluation expands the scope for ex post adjustment of formal rules (Alonso & Andrews, 2016), making execution credibility more dependent on governance structures and amplifying the moderating role of family ownership. Based on the preceding discussion, our final hypothesis is as follows:

#### Hypothesis 4 (H4)

The negative moderating effect of family ownership is stronger for SMEs and service-sector firms.

## Empirical strategy

### Data sources and the sample

The data on structured management practices comes from the Management and Expectation Survey (MES), collected by the UK Office for National Statistics (ONS) (Office for National Statistics, 2025). This survey is very similar to well-established management practices surveys conducted for other developed countries (Bloom et al., 2013, 2019) for the USA; (Broszeit et al., 2019) for Germany).^3^ Compared to those surveys, the MES has two main advantages. First, it includes small establishments/firms with ten or more employees, offering insights into the management practices of small businesses. Second, it covers a wider range of industries, spanning from manufacturing to services sectors. The first wave of the MES refers to 2016, while the second refers to 2020. The MES 2020 includes questions relating to 2019 and 2020. Therefore, these two waves provide data for three years: 2016, 2019, and 2020.

The productivity data comes from the Annual Respondents Database X (ARDx),^4^ maintained by the ONS (Office for National Statistics, 2023). The ARDx dataset covers 2008–2020 years and contains fewer firms than the MES. We link the ARDx with the MES using a unique firm identifier. All observations from the ARDx and 52% of observations from the MES are merged, resulting in 16,340 valid observations for 2016, 2019, and 2020.^5^ The merged dataset covers 73 industries classified under 2-digit Standard Industrial Classification (SIC) codes and 11 first level International Territorial Level (ITL1) regions/nations across Great Britain (excluding Northern Ireland).

Table 6 in the Appendix reports the distribution of observations by year. We observe a decline in the number of observations over time, which may reflect reduced availability of capital stock data in the ARDx dataset. Table 7 in the Appendix compares sample distribution of the MES and the merged MES-ARDx datasets by firm characteristics across family-owned and non-family-owned firms. After merging the composition of two datasets remains largely consistent.

### Model specification

To examine the relationship between structured management practices and productivity, and to assess how family ownership moderates this relationship, we follow Jirjahn et al. (2024) and adopt a within-between random effects (Mundlak) model (Bell et al., 2019; Mundlak, 1978). This approach allows us to separately identify within-firm and between-firm effects of management practices. Compared with pooled OLS and fixed effects or random effects panel data estimators, this method is more appropriate in our setting, which features a relatively short panel with a limited number of observations per firm. The relationship between management practices and firm productivity is subject to endogeneity, as more productive firms may be more likely to adopt, formalise, or sustain structured management practices, rather than management practices causing higher productivity. It could be that high-productivity firms have more resources to invest in professional management systems or may face stronger pressure from investors, lenders, or regulators to adopt formal practices. This can lead to an upward bias in estimated productivity returns to management practices, especially in cross-sectional or between-firm comparisons. The estimation of within-firm effects in Mundlak model controls for time-invariant unobserved firm heterogeneity that may be correlated with management practices, thereby mitigating endogeneity concerns between management practices and productivity. At the same time, it enables the estimation of between-firm effects, reflecting cross-sectional differences, which however, could be biased upward.

Following Jirjahn et al. (2024) and Bell et al. (2019), we decompose time-varying controls (${x}_{it}$) into firm-specific averages (${\overline{x} }_{i}$) and within-firm deviations from these averages ${(x}_{it}-{\overline{x} }_{i})$, allowing simultaneous estimation of within-firm and between-firm effects (a reformulated Mundlak model). The baseline specification is as follows:

1 $$y_{it}=\beta_0+{\beta'}_1\left(x_{it}-{\overline x}_i\right)+{\beta'}_2{\overline x}_i+{\beta'}_3z_i+u_i+f_s+\gamma_r+\tau_t+\varepsilon_{it}$$

where ${y}_{it}$ is the natural log of labour productivity of firm $i$ in year $t$; ${x}_{it}$ is a vector of time-varying firm characteristics including the management practices score; ${\overline{x} }_{i}$ is a vector of time-varying firm characteristics averaged over all observed time periods; ${z}_{i}$ is a vector of time-invariant firm characteristics including family ownership; ${u}_{i}$ captures firm-specific random effects; ${f}_{s}$, ${\gamma }_{r}$, ${\tau }_{t}$ denote sector, region, and year fixed effects, respectively; and ${\varepsilon }_{it}$ is the idiosyncratic error term.

To examine whether family ownership (${F}_{i}$) can moderate the relationship between management practices (${M}_{it}$) and productivity, we augment specification (1) with two interaction terms: $\left({M}_{it}-{\overline{M} }_{i}\right)\times {F}_{i}$ and ${\overline{M} }_{i}\times {F}_{i}$. Equation (1) is estimated using random effects model, with clustered standard errors at the establishment level.

The vector of coefficients ${\beta {\prime}}_{1}$ captures the within-firm effects of time-varying variables on productivity, which is equivalent to the estimator from a fixed effects model. The vector of coefficients ${\beta {\prime}}_{2}$ reflects the between-firm effects of time-varying variables on productivity coming from cross-sectional average differences. The within-firm estimators can be interpreted as ‘short-run’ effects on productivity, unbiased, as they are less subject to endogeneity. Under the assumption of no omitted firm-level variables, the between-firms estimators are also unbiased and can be interpreted as the ‘long-run’ effects. To ensure the validity of this assumption, and to reduce the bias, we control for a rich set of fixed effects and firm characteristics, including capital per worker, employment, family and foreign ownership, export status, company legal status, and education of managers and workers. All variables are described in the next section and in Table 10 of the Appendix.

### Variables

#### The management practices score

The MES contains 16 questions related to the structured management practices scores. Each question is a multiple choice, where the response options are coded with scores on a scale from 0 (the management practice is absent) to 1 (the management practice is high). Table 26 in the Appendix lists all questions, responses and corresponding scores. To construct an overall management practice score, we follow Bloom and Van Reenen (2007), Bloom et al. (2019), and Broszeit et al. (2019), and compute an unweighted average of 16 questions. As a result, it ranges from 0 to 1, where 0 indicates the lowest intensity of structured management practices and 1 indicates its highest intensity.

To assess heterogeneity across management dimensions and test hypothesis H3a, we divide 16 questions into four groups to construct sub-scores: monitoring, targets, incentives, and training (Bloom & Van Reenen, 2007; Broszeit et al., 2019). The monitoring score is calculated as the average of questions 1 to 4, capturing problem solving practices, the number of key performance indicators, and the frequency of performance reviews for managers and non-managers. The targets score is computed as the average of questions 5 to 8, which cover the main timeframe of targets, the difficulty of achieving targets, and the extent to which targets are communicated to managers and non-managers. The incentives score is defined as the average of questions 9 to 12 and reflects the basis for performance-related bonuses and promotion criteria for managers and non-managers. The training score is calculated as the average of questions 13 and 14 and measures the average number of training and development days undertaken by managers and non-managers.^6^

In addition, to test hypothesis H3b, we distinguish between management practices targeted at managers and those targeted at non-managerial employees and construct two corresponding indices. The manager-focused score is calculated as the average of questions 3, 7, 9, 11, and 13, while the non-manager-focused score is based on questions 4, 8, 10, 12, and 14.

For robustness checks, we also construct alternative management practices measures based on *Z*-score standardisation of unweighted average of all 16 questions, an unweighted average score excluding questions 15 and 16, and a first principal component derived from all 16 management practice questions. Summary statistics for individual questions and all variations of management practice scores are reported in Table 8. Table 9 shows the summary statistics of management practices score across different categories, for family and non-family firms, based on the MES sample and the merged MES-ARDx sample. It is noticeable that family firms have lower management practices scores across all dimensions than non-family firms.

#### Other variables

The main dependent variable is labour productivity, measured as gross value added per worker, which comes from the ARDx. Gross value added at basic prices is approximated as output at basic prices minus intermediate consumption at purchasers’ prices, following Office for National Statistics (2023).

Time-varying firm-level variables include the number of employees and capital stock per worker. Both gross value added and capital stock are deflated using two-digit SIC industry-level GDP deflators provided in ARDx, with 2019 as the base year. Firm age is also included as a time-varying control variable.

Time-invariant firm characteristics are defined using an ‘ever-equals-one’ rule over the sample period. This choice reflects the short time dimension of our panel, the limited number of observations per firm, and the slow-moving nature of these characteristics. This approach is consistent with standard practice in short-panel settings and helps mitigate misclassification arising from measurement error (Bell et al., 2019; Mundlak, 1978; Wooldridge, 2010). It also reduces possible endogeneity between these firm characteristics and changes in productivity.

The family ownership indicator is based on the MES survey question, ‘Which of the following applies to your business’s ownership structure?’ Firms reporting a family-owned structure are coded as 1 and 0 otherwise. For the subsample of family-owned firms, we construct indicators for family management and non-family management based on the question, ‘Did the managing director or equivalent have any form of family connection or relationship with the business owners?’ The family management (non-family management) indicator equals 1 if the response is ‘yes’ (‘no’). These variables allow us to distinguish between family-managed firms and family-owned firms with non-family professional managers, and to test whether these management structures have different moderating effects.

Export status is coded as 1 if the firm reports exporting activity and 0 otherwise. Foreign ownership is defined as ultimate ownership held by non-UK owners. Company status is coded as 1 if the firm is a limited company and 0 otherwise. Table 10 reports the definitions and data sources of all variables in our analysis, while Table 11 presents the summary statistics.

## Empirical findings

### Baseline results

Table 1 reports the baseline results^7^ from the estimation of specification (1), in which the effect of management practices on productivity is decomposed into within-firm and between-firm components using the reformulated Mundlak approach. The within-firm effect captures the short-run impact of increase in the management practices score within firms over the sample period (2016, 2019, and 2020) on labour productivity, while the between-firm effect reflects long-run association between management practices and productivity.

**Table 1:** Baseline results

Dependent variable: ln(GVA per worker)						
	(1)		(2)		(3)	
	Within	Between	Within	Between	Within	Between
Score	0.472***	0.728***	0.477**	1.071***	0.568***	1.078***
	(0.108)	(0.050)	(0.195)	(0.099)	(0.185)	(0.095)
Score × family ownership			0.001	− 0.472***		
			(0.231)	(0.106)		
Score × family management					− 0.220	− 0.458***
					(0.223)	(0.104)
Score × non-family management					0.076	− 0.513***
					(0.277)	(0.126)
Family ownership		− 0.091***		0.185***		
		(0.019)		(0.067)		
Family management						0.180***
						(0.065)
Non-family management						0.248***
						(0.077)
Controls	Yes		Yes		Yes	
Industry FE	Yes		Yes		Yes	
Region FE	Yes		Yes		Yes	
Year FE	Yes		Yes		Yes	
*N* of observations	16,340		16,340		16,340	
*N* of clusters	12,010		12,010		12,010	
Overall *R*-sq	0.320		0.322		0.322	

The dependent variable is ln(GVA per worker). All specifications are estimated using a reformulated Mundlak model. Time-varying control variables include the natural logarithm of the number of employees, the natural logarithm of capital stock per worker, and the natural logarithm of firm age. Time-invariant firm characteristics include company legal status, exporter status, foreign ownership, managers’ education, and workers’ education. All specifications include industry, region, and year fixed effects. Standard errors are clustered at the establishment level and reported in parentheses. The full set of coefficient estimates is reported in Table 12 in the Appendix. *, **, and *** denote statistical significance at the 10%, 5%, and 1% levels, respectively

Source: Authors’ own calculations using ONS ARDx and MES surveys

Column (1) of Table 1 tests our first hypothesis (H1) and shows a positive and statistically significant relationship between management practices score and labour productivity for both the within-firm and between-firm components.^8^,^9^ The within-firm estimate implies that a 0.1 increase in the management practices score within a firm is associated with an approximately 4.8% increase in GVA per worker. This suggests that improvements in management practices positively impact labour productivity even over a relatively short period of time. By contrast, the larger between-firm coefficient suggests that firms with higher management quality exhibit higher productivity levels. A sustained 0.1 higher management score is associated with around a 7.6% higher labour productivity,^10^ highlighting the importance of management practices as a long-run organisational capability. To examine whether the relationship between management practices and productivity varies by family ownership, we augment the baseline specification (1) by introducing an interaction term between the management practices score and a family ownership dummy variable. The results reported in column (2) of Table 1 support our second hypothesis (H2) and indicate that family ownership significantly weakens the between-firm effect of management practices on productivity, while it does not change the within-firm effect. Economically, the long-run marginal effect of management practices for family-owned firms is reduced to 0.599 (1.071–0.472), which is 17.8% lower than the baseline between-firm estimate of 0.728 reported in column (1). The results suggest that family ownership shapes the organisational conditions under which management practices are implemented. While short-run improvements in management practices are associated with immediate productivity gains, long-run productivity returns depend on the consistent application of formal management systems over time and may be undermined by greater flexibility and discretion in the enforcement of formal rules in family firms.

**Fig. 1:**
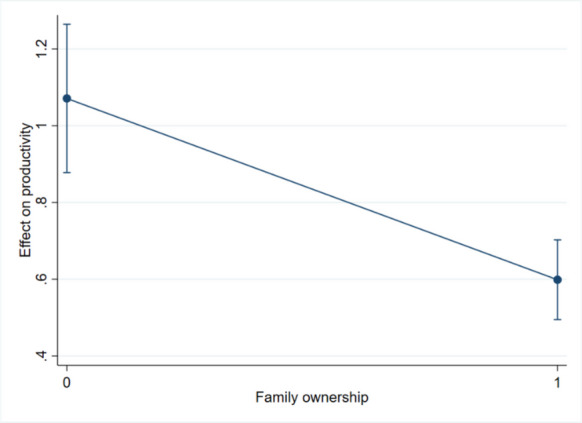
Average marginal effects of management practices score (between-firm) on productivity by family ownership. Notes: This figure reports the marginal effect of between-firm management practices on labour productivity, moderated by family ownership. Estimates are based on column (2) of Table B1. Vertical bars denote 95% confidence intervals. Source: Authors’ own calculations using ONS ARDx and MES surveys

**Fig. 2:**
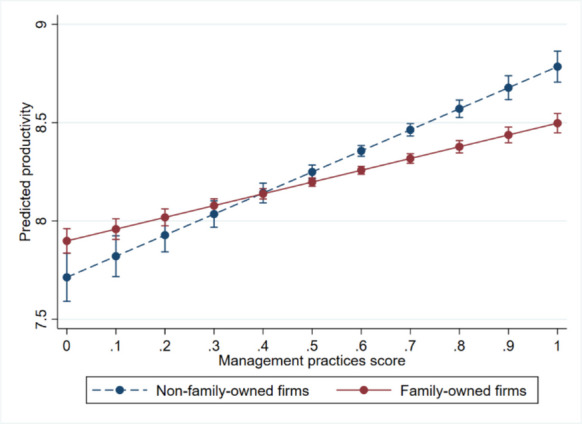
Predicted labour productivity by management practices score for family-owned and non-family-owned firms. Notes: This figure shows predicted values of ln(GVA per worker) over the management practices score for family-owned and non-family-owned firms. Predictions are computed from column (2) of Table B1, holding all other covariates at their sample means. Error bars represent 95% confidence intervals. Source: Authors’ own calculations using ONS ARDx and MES surveys

Figure 1 in the Appendix visualises the moderating role of family ownership, showing that the long-run (between-firm) productivity returns to management practices are lower for family firms than for non-family firms. Figure 2 further illustrates that, while higher management practices scores are associated with higher predicted labour productivity for both ownership types, the relationship is flatter for family-owned firms, implying smaller productivity gains from improvements in management practices.

To investigate heterogeneity in the organisational structure of family-owned firms, we distinguish between family firms managed by a family member and those managed by a non-family professional. We then augment specification (1) by introducing two interaction terms between management practices score and these alternative management structures. The results reported in column (3) of Table 1 show that irrespective of management structure, family firms exhibit a weaker between-firm relationship between management practices and productivity compared with non-family firms. Although the moderating effect is more negative for family firms managed by non-family professionals, it is not statistically different from the corresponding effect for family-managed firms.^11^ The interactions with the within-firm component are statistically insignificantly for both management structures, as in column (2). These findings suggest that the attenuation of long-run (between-firm) productivity returns to management practices reflect structural features of family ownership, such as governance arrangements, incentive structures, and persistent organisational constraints, rather than differences in managerial control (family versus non-family management).

### Robustness checks

To assess the robustness of our baseline finding that family ownership weakens the productivity returns to management practices, we conduct a series of robustness checks using alternative estimation methods, construction of the management practices score, measures of firm performance, and an instrumental-variable approach.

Table 13 in the Appendix reports the results from three alternative model specifications. Column (1) presents estimates from a random effects model, column (2) reports ordinary least squares (OLS) estimates, and column (3) applies the Levinsohn and Petrin (2003) production-function approach while allowing for a moderating role of family ownership. Across all specifications, the interaction between the management practices score and family ownership remains negative and statistically significant. As expected, the estimates are close to the between-firm effects reported in column (2) of Table 1.

Table 14 in the Appendix examines robustness to alternative construction of the management practices score. We consider three distinct measures: an unweighted average of questions 1–14 (Score-14), a standardised unweighted average management practices score (*Z*-score), and a first principal component derived from all 16 management practice questions (PCA score). The results remain highly consistent: the management practices score is positively associated with productivity, while its interaction with family ownership is negative and statistically significant for the between-firm component, but statistically insignificant for the within-firm component.

Table 15 in the Appendix assesses robustness to alternative measures of firm performance. Following Bender et al. (2018), in column (1), we use total factor productivity (TFP) as dependent variable.^12^ In column (2), we follow Bloom and Van Reenen (2007) by using output per worker as the dependent variable and additionally controlling for intermediate inputs per worker in the regression. To address potential sample selection concerns arising from merging ARDx and MES data, column (3) relies on the original MES dataset and uses turnover per worker (directly provided by the MES) as the outcome variable. Although data coverage and control variables differ across all three specifications, the moderating pattern of family ownership remains unchanged.

Table 16 in the Appendix reports the results from a specification, where we add interaction terms of all independent variables with family ownership indicator. In this way, we allow the characteristics of family-owned firms to be different from non-family-owned firms. The results remain robust and consistent with our baseline finding. Moreover, almost all additional interaction terms between family ownership and other firm characteristics are statistically insignificant, apart from the within-firm effect of capital per worker and the between-firm effect of managers’ education. This indicates that, in the MES-ARDx dataset, observable characteristics of family firms are broadly comparable to those of non-family firms, and that the differential productivity returns to management practices are not driven by systematic differences in observed firm attributes.

Finally, Table 17 in the Appendix addresses potential endogeneity concerns between management practices and productivity using a two-stage least squares (2SLS) approach within the reformulated Mundlak framework. Following Fisman and Svensson (2007), in column (1), the instrument is the management practices score averaged at the two-digit industry, region, and survey wave level. This average and its interaction with the family ownership dummy variable are used as instruments for the management practice score and its interaction with family ownership dummy variable. Following Cornett et al. (2007), in column (2), the instrument is one-period lag of within-firm management practices score. While 2SLS approach helps mitigate reverse causality issues, we acknowledge that our instruments are not completely exogeneous. Across both specifications, the negative moderating effect of family ownership on the long-run productivity returns to management practices remains statistically significant. Our findings therefore are consistent with experimental causality evidence showing that improvements in management practices can raise productivity (Bloom et al., 2012a).

### Heterogeneity in the execution of structured management practices

This subsection examines the moderating effect of family ownership by decomposing the aggregate management practices score into sub-dimensions.

Table 2 reports the results for the four dimensions of management practices: monitoring, targets, incentives, and training. The estimates indicate that the long-run productivity returns to all four dimensions are weakened by family ownership. The magnitudes of the moderating effects, however, vary across dimensions. The between-firm interaction between family ownership and incentives-related managerial practices score is negative and larger than those associated with target setting, followed by monitoring and training. This result is consistent with hypothesis H3a and suggests that, in family firms, management practices that rely on credible performance-based incentives and binding target commitments, translate less effectively into productivity gains. A plausible explanation is that stronger informal control and discretionary interventions by family owners undermine the credibility of formal incentive and target-setting systems, while practices such as monitoring and training are less sensitive to such governance frictions.

**Table 2:** Sub-scores analysis

Dependent variable: ln(GVA per worker)								
	(1)		(2)		(3)		(4)	
	Within	Between	Within	Between	Within	Between	Within	Between
Monitoring	0.400**	0.583***						
	(0.174)	(0.094)						
Monitoring × family ownership	− 0.243	− 0.194*						
	(0.202)	(0.104)						
Targets			0.171	0.431***				
			(0.109)	(0.070)				
Targets × family ownership			− 0.037	− 0.207***				
			(0.124)	(0.077)				
Incentives					0.163*	0.668***		
					(0.097)	(0.056)		
Incentives × family ownership					0.075	− 0.249***		
					(0.121)	(0.065)		
Training							0.183	0.397***
							(0.119)	(0.065)
Training × family ownership							0.089	− 0.149**
							(0.141)	(0.074)
Family ownership		0.018		0.043		0.022		− 0.021
		(0.065)		(0.054)		(0.038)		(0.047)
Controls	Yes		Yes		Yes		Yes	
Industry FE	Yes		Yes		Yes		Yes	
Region FE	Yes		Yes		Yes		Yes	
Year FE	Yes		Yes		Yes		Yes	
*N* of observations	16,340		16,340		16,340		16,340	
*N* of clusters	12,010		12,010		12,010		12,010	
Overall *R*-sq	0.312		0.312		0.321		0.312	

The dependent variable is ln(GVA per worker). All specifications are estimated using a reformulated Mundlak model. Columns (1)–(4) report the results for different dimensions of management practices. Monitoring is constructed as the average score of survey questions Q1–4, targets as the average score of Q5–8, incentives as the average score of Q9–12, and training as the average score of Q13–14. Time-varying control variables include the natural logarithm of the number of employees, the natural logarithm of capital stock per worker, and the natural logarithm of firm age. Time-invariant firm characteristics include company legal status, exporter status, foreign ownership, managers’ education, and workers’ education. All specifications include industry, region, and year fixed effects. Standard errors are clustered at the establishment level and reported in parentheses. *, **, and *** denote statistical significance at the 10%, 5%, and 1% levels, respectively

Source: Authors’ own calculations using ONS ARDx and MES surveys

We also distinguish management practices targeted to managers and to non-managers. As shown in Table 3, the interaction terms (between-firm effects) in columns (1) and (2) are negative and statistically significant. The moderating effect of family ownership is larger for management practices targeted at managers than for those targeted at non-managerial workers. This finding supports our hypothesis H3b and is consistent with an execution-based property rights theory of family firms. Manager-oriented practices directly constrain decision authority and rely more heavily on credible commitment in enforcement, which is more likely to be undermined in family-owned firms where residual control rights permit discretionary intervention. In turn, worker-oriented managerial practices are less exposed to such execution distortions.

**Table 3:** Sub-scores analysis: targeting managers and non-managers

Dependent variable: ln(GVA per worker)				
	(1)		(2)	
	Within	Between	Within	Between
Managers’ score	0.429***	0.953***		
	(0.151)	(0.088)		
Managers’ score × family ownership	− 0.095	− 0.435***		
	(0.178)	(0.096)		
Workers’ score			0.321*	0.873***
			(0.166)	(0.079)
Workers’ score × family ownership			0.134	− 0.341***
			(0.196)	(0.090)
Family ownership		0.148**		0.068
		(0.058)		(0.048)
Controls	Yes		Yes	
Industry FE	Yes		Yes	
Region FE	Yes		Yes	
Year FE	Yes		Yes	
*N* of observations	16,340		16,340	
*N* of clusters	12,010		12,010	
Overall *R*-sq	0.32		0.32	

The dependent variable is the natural logarithm of GVA per worker. All specifications are estimated using a reformulated Mundlak model. Managers’ score is constructed as the average score of survey questions 3, 7, 9, 11, 13, 15, while workers’ score is constructed as the average score of 4, 8, 10, 14, 16. Time-varying control variables include the natural logarithm of the number of employees, the natural logarithm of capital stock per worker, and the natural logarithm of firm age. Time-invariant firm characteristics include company legal status, exporter status, foreign ownership, managers’ education, and workers’ education. All specifications include industry, region, and year fixed effects. Standard errors are clustered at the establishment level and reported in parentheses. *, **, and *** denote statistical significance at the 10%, 5%, and 1% levels, respectively

Source: Authors’ own calculations using ONS ARDx and MES surveys

### Firm size and industry heterogeneity in execution credibility of structured management practices

Table 4 examines whether the moderating role of family ownership varies across firm sizes and industry sub-samples. Columns (1) and (2) of Table 4 report estimates for SMEs and large firms, while columns (3) and (4) distinguish between manufacturing and service sectors. SMEs are firms with less than 250 employees. Manufacturing sector is defined by 2-digit SIC codes ranging from 10 to 33. Services sector is defined by 2-digit SIC codes 45–96. We exclude the water supply, sewerage, waste management and remediation (codes 36–39) industries, as well as the construction (codes 41–43) industry, from the service sector because they are more regulated and dependent on the public sector.

**Table 4:** Moderating effects of family ownership by sub-groups

Dependent variable: ln(GVA per worker)								
	(1)		(2)		(3)		(4)	
	SMEs		Large		Manufacturing		Services	
	Within	Between	Within	Between	Within	Between	Within	Between
Score	0.625**	1.118***	0.378	0.844***	0.447*	0.562***	0.460*	1.259***
	(0.251)	(0.113)	(0.304)	(0.217)	(0.261)	(0.146)	(0.259)	(0.131)
Score × family ownership	− 0.120	− 0.532***	0.007	− 0.170	− 0.286	− 0.193	0.182	− 0.571***
	(0.285)	(0.121)	(0.407)	(0.311)	(0.352)	(0.164)	(0.308)	(0.141)
Family ownership		0.212***		0.015		0.052		0.245***
		(0.073)		(0.221)		(0.105)		(0.090)
Controls	Yes		Yes		Yes		Yes	
Industry FE	Yes		Yes		Yes		Yes	
Region FE	Yes		Yes		Yes		Yes	
Year FE	Yes		Yes		Yes		Yes	
*N* of observations	13,032		3308		4214		10,560	
*N* of clusters	9806		2323		2849		7965	
Overall *R*-sq	0.304		0.413		0.170		0.333	

The dependent variable is the natural logarithm of GVA per worker. Columns (1)–(4) report estimates for subsamples of SMEs, large firms, manufacturing, and services sector, respectively. All specifications are estimated using a reformulated Mundlak model. Large firms are defined as firms with 250 employees or more. Manufacturing is defined as 2-digit SIC code from 10 to 33. Services section is defined as 2-digit SIC code from 45 to 96. Time-varying control variables include the natural logarithm of the number of employees, the natural logarithm of capital stock per worker, and the natural logarithm of firm age. Time-invariant firm characteristics include company legal status, exporter status, foreign ownership, manager’s education, and worker’s education. All specifications include industry, region, and year fixed effects. Standard errors are clustered at the establishment level and reported in parentheses. *, **, and *** denote statistical significance at the 10%, 5%, and 1% levels, respectively

Source: Authors’ own calculations using ONS ARDx and MES surveys

According to columns (1) and (2), the long-run (between-firm) productivity returns to management practices are substantially lower for family-owned SMEs relative to their non-family counterparts. By contrast, family ownership does not alter the productivity implications of management practices in larger organisations.

A similar asymmetry is observed across industries. In the service sector, family ownership significantly attenuates the long-run productivity returns to management practices, as reflected by a negative and statistically significant between-firm interaction coefficient. In the manufacturing sector, however, the interaction term is statistically insignificant, suggesting that family ownership does not play a meaningful moderating role in shaping the productivity effects of management practices in this sector. These findings confirm our last hypothesis H4, discussed in the theoretical framework Section 2.3, which emphasise the role of execution credibility and residual control rights in shaping the productivity returns to management practices.

Tables 18 and 19 in the Appendix further explore the heterogeneity of effects by focusing on different characteristics of firms. In Table 18, we distinguish between knowledge-intensive and low-skilled services.^13^ The results show that the negative moderating role of family ownership is present in both types of services, but particularly pronounced in knowledge-intensive services.

Table 19 in the Appendix further explores the heterogeneity of moderating effects by looking at SMEs services, SMEs manufacturing, large firms services, SMEs knowledge-intensive services, and SMEs low-skilled services. We notice the interaction terms are statistically insignificant in manufacturing SMEs and large firms in the services sector. By contrast, the negative moderating effect of family ownership is concentrated in service-oriented SMEs, where the interaction term is both economically large and statistically significant. This effect is particularly pronounced in knowledge-intensive service industries, in which family ownership not only attenuates the long-run (between-firm) effect, but also the short-run (within-firm) effect productivity returns to structured management practices. The stronger negative moderating effect observed in knowledge-intensive services relative to low-skilled services is consistent with our execution-credibility framework. In knowledge-intensive settings, productivity depends more heavily on intangible inputs, discretionary effort, and credible performance-based incentives (Cette et al., 2024), making formal management systems particularly sensitive to discretionary enforcement under family ownership. In contrast, routinised tasks and higher observability in low-skilled services reduce the productivity consequences of weakened execution credibility.

## Conclusion

This paper examines how family ownership moderates the relationship between structured management practices and firm productivity. Using merged data from the UK Management and Expectations Survey (MES) and the Annual Respondents Database X (ARDx), we apply a reformulated Mundlak framework to decompose the productivity effects of management practices into short-run (within-firm) and long-run (between-firm) components. The results show that management practices are positively associated with labour productivity on average, but that this association is significantly weaker in family-owned firms. More specifically, the moderating effect of family ownership operates mainly through the long-run effect, while differences in short-run within-firm effects are not statistically significant. These results are robust to using alternative specifications, dependent and independent variables. We also find that the negative moderating effect of family ownership persists regardless of whether firms are managed by family members or by non-family professional managers. This suggests that the moderating role of family ownership reflects broader governance conditions rather, than family involvement in firm management alone.

Our results are supported by the property rights theory of the family firm, when residual control rights under incomplete contracting shape the credibility of execution of organisational arrangements. This, in turn, affects the consistent enforcement of targets, performance evaluation, and incentive realisation. Align with this argument, we observe heterogeneous moderating effects across different dimensions of management practices. Family ownership exerts a stronger attenuating effect on practices that rely most heavily on performance commitments and allocation rules, such as incentive schemes and target setting. The moderating effects of family ownership are also larger for practices targeted at managers than at non-managerial workers.

The moderating role of family ownership is also more pronounced among SMEs and in the service sector. These contexts are typically characterised by weaker formal governance structures, greater difficulty in verifying performance outcomes, and a larger scope for discretionary intervention. As a result, the execution of formal management systems is more fragile, and the long-run productivity returns to management practices are more easily eroded.

The policy implications of our findings suggest that productivity improvements should not rely solely on encouraging firms to adopt a greater number of structured management tools. Instead, the effectiveness of such tools depends critically on whether they operate within a stable, predictable, and enforceable execution environment. For family firms in particular, in the absence of adequate governance and execution infrastructure, the simple introduction of KPIs, targets, or incentive schemes may fail to deliver the expected long-run returns. A more reliable approach is therefore to combine management improvement with governance improvement. Strengthening human resource processes, improving the verifiability of performance evaluation, limiting discretionary exceptions, and enhancing transparency and accountability can help establish execution credibility. Once these foundations are in place the long-run organisational returns to management practices can be fully realised. For SMEs and service firms, this is particularly important.

This study is subject to several limitations. First, we emphasise strong correlational evidence supported by causal robustness checks rather than making definitive causal claims. Future research therefore could exploit more clearly exogenous shocks to identify the causal mechanisms linking family governance and management practices, such as succession events, transitions from family to professional management, or policy-induced quasi-natural experiments using difference-in-differences designs. Second, family ownership and managerial structure are measured primarily using binary indicators, which limits our ability to capture more detailed dimensions of governance heterogeneity, such as ownership concentration, generational stage, or the depth of family involvement in management. Further progress may also be achieved by incorporating richer measures of governance intensity and non-economic objectives, which would help clarify the boundary conditions under which execution discretion translates into weaker management effectiveness.

## Data Availability

The data used are confidential and available from the UK Office for National Statistics Secure Research Service. The DOI references for the data used are 10.5255/UKDA-SN-7989-5 and 10.5255/UKDA-SN-8557-6.

## Appendix

**Table 5:** Data cleaning and sample construction

Sample	Remaining observations
MES 2016 (raw)	24,998
MES 2016 (valid responses)	9501
MES 2020 (raw)	14,658
MES 2020 (valid responses)	12,414
MES 2020 reshaped (2019 and 2020)	24,828
Pooled MES sample (2016, 2019, 2020)	34,329
All 16 management practices questions answered	32,403
Consistent family ownership information	32,385
Stable ownership status	31,569
Matched with ARDx	17,569
Matched sample with missing productivity data	1229
Final estimation sample (MES-ARDx)	16,340

This table reports the sample construction and data cleaning process. The Management and Expectations Survey (MES) was conducted in 2016 and 2020, with the 2020 wave additionally collecting retrospective information for 2019. After retaining firms with valid survey responses and complete answers to all 16 management practice questions, we exclude observations with inconsistent or time-varying ownership status over the sample period. The MES data are then merged with the UK Annual Respondents Database (ARDx) to obtain firm-level productivity measures. Observations without valid productivity information or without a successful match to ARDx are dropped. The final sample consists of 16,340 firm-year observations used in the regression analysis

**Table 6:** The distribution of observations by year

Year	MES	MES-ARDx	%
2016	7294	5576	76%
2019	12,138	6322	52%
2020	12,137	4442	37%
Total	31,569	16,340	52%

This table reports the number of observations by survey year. MES denotes the Management and Expectations Survey sample. MES-ARDx refers to the subsample of observations from the MES matched to the ARDx administrative data using firm identifiers. The percentage column reports the share of MES observations that are merged in each year. The final row reports total number of observations

Source: Authors’ own calculations using ONS ARDx and MES surveys

**Table 7:** Sample distribution by sub-groups of firms

	Family-owned	Non-family-owned	Overall	% of overall
	(1)	(2)	(3)	(4)
***MES***				
Overall	19,946 (63%)	11,623 (37%)	31,569	100%
SMEs	18,190 (67%)	8817 (33%)	27,007	86%
Large	1756 (38%)	2806 (62%)	4562	14%
Foreign	913 (25%)	2686 (75%)	3599	11%
Domestic	19,033 (68%)	8937 (32%)	27,970	89%
Family management	13,768 (100%)	0	13,768	44%
Non-family management	6178 (100%)	0	6178	20%
Manufacturing	4212 (65%)	2252 (35%)	6464	20%
Services	13,313 (61%)	8590 (39%)	21,903	69%
Knowledge intensive	5097 (49%)	5250 (51%)	10,347	33%
Low-skilled	8216 (71%)	3340 (29%)	11,556	37%
***MES-ARDx***				
Overall	10,047 (61%)	6293 (39%)	16,340	100%
SMEs	8755 (67%)	4277 (33%)	13,032	80%
Large	1292 (39%)	2016 (61%)	3308	20%
Foreign	580 (24%)	1812 (76%)	2392	15%
Domestic	9490 (68%)	4481 (32%)	13,948	85%
Family management	7148 (100%)	0	7148	44%
Non-family management	2899 (100%)	0	2899	18%
Manufacturing	2627 (62%)	1587 (38%)	4214	26%
Services	6233 (59%)	4327 (41%)	10,560	65%
Knowledge intensive services	2180 (47%)	2466 (53%)	4646	28%
Low-skilled services	4053 (69%)	1861 (31%)	5914	36%

This table presents the sample distribution across firm sub-groups for the MES sample and the merged MES–ARDx sample. Percentages in column (4) are computed relative to the total number of observations within each panel. In columns (1) and (2), the percentages in parentheses represent the share of observations in that column relative to the total in column (3)

Source: Authors’ own calculations using ONS ARDx and MES surveys

**Table 8:** Summary statistics for management practices score

Variable	Mean	Std. Dev	Mean	Std. Dev
	MES		MES-ARDx	
***Monitoring***				
Score_Q1	0.813	0.227	0.821	0.216
Score_Q2	0.633	0.310	0.666	0.300
Score_Q3	0.431	0.231	0.455	0.225
Score_Q4	0.290	0.278	0.312	0.284
***Targets***				
Score_Q5	0.579	0.368	0.594	0.362
Score_Q6	0.665	0.358	0.688	0.344
Score_Q7	0.744	0.366	0.758	0.348
Score_Q8	0.477	0.354	0.480	0.342
***Incentives***				
Score_Q9	0.315	0.355	0.349	0.359
Score_Q10	0.296	0.380	0.322	0.385
Score_Q11	0.546	0.437	0.580	0.431
Score_Q12	0.615	0.417	0.651	0.404
***Training***				
Score_Q13	0.541	0.294	0.545	0.284
Score_Q14	0.553	0.287	0.561	0.276
***Other***				
Score_Q15	0.561	0.480	0.595	0.472
Score_Q16	0.706	0.438	0.745	0.416
***Overall scores***				
Score	0.548	0.200	0.570	0.192
Score14	0.536	0.193	0.556	0.185
*Z*-score	0.000	1.000	0.095	0.957
PCA score	0.000	2.289	0.216	2.195
***Sub-scores***				
Monitor	0.542	0.197	0.563	0.191
Targets	0.616	0.290	0.630	0.275
Incentive	0.443	0.291	0.475	0.286
Training	0.547	0.268	0.553	0.257
Managers’ score	0.515	0.213	0.537	0.206
Workers’ score	0.446	0.212	0.465	0.206
*N* observations	31,569		16,340	

This table reports summary statistics for management practices scores for the MES sample and the merged MES–ARDx sample. Score_Q1 to Score_Q16 correspond to individual MES management practice questions. Score is the unweighted average of all 16 questions, while Score14 is the unweighted average of questions Q1–Q14. *Z*-score is the standardised unweighted management practices score. PCA score is the first principal component extracted from the full set of management practice questions. The managers’ score is defined as the average of questions 3, 7, 9, and 13, while the workers’ score is based on questions 4, 8, 10, and 14

Source: Authors’ own calculations using ONS ARDx and MES surveys

**Table 9:** Summary statistics for management practices score by groups

	Overall			Family-owned			Non-family-owned		
	Obs	Mean	Std	Obs	Mean	Std	Obs	Mean	Std
***MES***									
Overall	31,569	0.548	0.200	19,946	0.522	0.206	11,623	0.592	0.180
SMEs	27,007	0.528	0.201	18,190	0.510	0.206	8817	0.564	0.183
Large	4562	0.667	0.148	1756	0.645	0.161	2806	0.681	0.137
Family owned	19,946	0.522	0.206	19,946	0.522	0.206	-	-	-
Non-family owned	11,623	0.592	0.180	-	-	-	11,623	0.592	0.180
Family management	13,768	0.512	0.204	-	-	-	-	-	-
Non-family management	6178	0.544	0.209	-	-	-	-	-	-
Manufacturing	6464	0.548	0.194	4212	0.515	0.196	2252	0.610	0.175
Services	21,903	0.553	0.200	13,313	0.529	0.209	8590	0.590	0.179
Knowledge intensive services	10,347	0.568	0.186	5097	0.550	0.200	5250	0.585	0.171
Low skilled services	11,556	0.539	0.210	8216	0.515	0.213	3340	0.597	0.191
***MES-ARDx***									
Overall	16,340	0.570	0.192	10,047	0.539	0.200	6293	0.619	0.167
SMEs	13,032	0.543	0.195	8755	0.522	0.201	4277	0.585	0.174
Large	3308	0.677	0.134	1292	0.655	0.151	2016	0.692	0.120
Family owned	10,047	0.539	0.200	10,047	0.539	0.200	-	-	-
Non-family owned	6293	0.619	0.167	-	-	-	6293	0.619	0.167
Family management	7148	0.526	0.201	7148	0.526	0.201	-	-	-
Non-family management	2899	0.571	0.195	2899	0.571	0.195	-	-	-
Manufacturing	4214	0.568	0.188	2627	0.531	0.193	1587	0.629	0.163
Services	10,560	0.577	0.190	6233	0.549	0.201	4327	0.618	0.165
Knowledge intensive services	4646	0.591	0.175	2180	0.569	0.190	2466	0.611	0.157
Low skilled services	5914	0.566	0.201	4053	0.538	0.205	1861	0.628	0.175

The management practices score is calculated as the unweighted average of 16 questions from the Management and Expectations Survey (MES). Manufacturing sectors are defined as industries with 2-digit SIC codes ranging from 10 to 33. Service sectors are defined as industries with 2-digit SIC codes from 45 to 96. Knowledge-intensive services include industries with 2-digit SIC codes 50, 51, 58–66, 69–75, 78, 80, and 84–88, as well as 90–93. All remaining service industries are classified as low-skilled services. SMEs are defined as firms with fewer than 250 employees, while large firms have 250 employees or more

Source: Authors’ own calculations using ONS ARDx and MES surveys

**Table 10:** Definitions of variables

Variable	Definition	Source
***Dependent variables***		
ln(GVA per worker)	Natural logarithm of gross value added per worker	ARDx
TFP	Total factor productivity estimated using the Levinsohn–Petrin method	ARDx
ln(output per worker)	Natural logarithm of output per worker	ARDx
ln(turnover per worker)	Natural logarithm of turnover per worker	MES
***Management practices score***		
Score	Unweighted average score across all 16 management practice questions	MES
Score14	Unweighted average score across questions 1–14	MES
*Z*-score	Standardised unweighted average score across all 16 management practice questions	MES
PCA score	A first principal component derived from all 16 management practice questions	MES
Monitor	Average score of monitoring practices (questions 1–4)	MES
Targets	Average score of target-setting practices (questions 5–8)	MES
Incentive	Average score of incentive and reward practices (questions 9–12)	MES
Training	Average score of training practices (questions 13–14)	MES
Managers’ score	Average score of managers-related questions (questions 3, 7, 9, 11, 13)	MES
Workers’ score	Average score of non-managers-related questions (questions 4, 8, 10, 12, 14)	MES
***Time-varying control variables***		
Material	Natural logarithm of intermediate material inputs	ARDx
Capital stock	Natural logarithm of capital stock per worker	ARDx
Firm age	Natural logarithm of firm age	MES
Employees	Natural logarithm of number of employees	MES
***Time invariant controls***		
Family ownership	Indicator equal to 1 if the firm is family-owned and 0 otherwise	MES
Foreign ownership	Indicator equal to 1 if the firm is foreign-owned based on ultimate ownership and 0 otherwise	MES
Family management	Indicator equal to 1 if the firm is reported as family-managed and 0 otherwise	MES
Non-family management	Indicator equal to 1 if the firm is family-owned but not family-managed and 0 otherwise	MES
Company	Indicator equal to 1 if the legal status is a limited liability company and 0 otherwise	MES
Export	Indicator equal to 1 if the firm engages in export activities and 0 otherwise	ARDx
Managers’ education	Indicator equal to 1 if more than 50% of managers hold a university degree and 0 otherwise	MES
Workers’ education	Indicator equal to 1 if more than 50% of non-managerial workers hold a university degree and 0 otherwise	MES

**Table 11:** Summary statistics of main variables

Variable	Obs	Mean	Std. Dev
***Dependent variables***			
ln(GVA per worker)	16,340	8.287	1.017
TFP	16,340	7.160	0.972
ln(output per worker)	16,340	4.539	1.203
ln(turnover per worker)	16,340	8.917	1.078
***Management practices score***			
Score	16,340	0.570	0.192
Score14	16,340	0.556	0.185
*Z*-score	16,340	0.095	0.957
PCA score	16,340	0.216	2.195
Monitor	16,340	0.563	0.191
Targets	16,340	0.630	0.275
Incentive	16,340	0.475	0.286
Training	16,340	0.553	0.257
Managers’ score	16,340	0.537	0.206
Workers’ score	16,340	0.465	0.206
***Time-varying control variables***			
Material	16,340	7.896	2.556
Capital stock	16,340	2.832	1.455
Firm age	16,340	2.915	0.621
Employees	16,340	4.331	1.289
***Time invariant controls***			
Family ownership	16,340	0.615	0.487
Foreign ownership	16,340	0.146	0.354
Family management	16,340	0.447	0.497
Non-family management	16,340	0.189	0.392
Company	16,340	0.902	0.297
Export	16,340	0.318	0.466
Managers’ education	16,340	0.364	0.481
Workers’ education	16,340	0.161	0.368

Summary statistics are based on the merged MES–ARDx sample. All continuous variables are in natural logarithms

Source: Authors’ own calculations using ONS ARDx and MES surveys

**Table 12:** Baseline results: all estimates

Dependent variable: Ln (GVA per worker)						
	(1)		(2)		(3)	
	Within	Between	Within	Between	Within	Between
Score	0.472***	0.728***	0.477**	1.071***	0.568***	1.078***
	(0.108)	(0.050)	(0.195)	(0.099)	(0.185)	(0.095)
Score × family ownership			0.001	-0.472***		
			(0.231)	(0.106)		
Score × family management					-0.220	-0.458***
					(0.223)	(0.104)
Score × non-family management					0.076	-0.513***
					(0.277)	(0.126)
Employees	-0.488***	-0.023***	-0.490***	-0.027***	-0.491***	-0.026***
	(0.078)	(0.008)	(0.078)	(0.008)	(0.078)	(0.008)
Capital stock	0.133***	0.173***	0.132***	0.171***	0.132***	0.171***
	(0.041)	(0.010)	(0.041)	(0.010)	(0.041)	(0.010)
Firm age	0.118**	0.082***	0.120**	0.081***	0.116**	0.082***
	(0.056)	(0.015)	(0.056)	(0.015)	(0.056)	(0.015)
Family ownership		-0.091***		0.185***		
		(0.019)		(0.067)		
Family management						0.180***
						(0.065)
Non-family management						0.248***
						(0.077)
Foreign ownership		0.161***		0.149***		0.149***
		(0.025)		(0.026)		(0.026)
Company		0.407***		0.405***		0.401***
		(0.037)		(0.037)		(0.037)
Export		0.133***		0.137***		0.137***
		(0.022)		(0.022)		(0.022)
Managers’ education		0.058***		0.056***		0.058***
		(0.020)		(0.020)		(0.020)
Workers’ education		0.164***		0.163***		0.163***
		(0.028)		(0.028)		(0.028)
Industry FE	Yes		Yes		Yes	
Region FE	Yes		Yes		Yes	
Year FE	Yes		Yes		Yes	
*N* of observations	16340		16340		16340	
*N* of cluster	12010		12010		12010	
Overall *R-sq*	0.320		0.322		0.322	

The dependent variable is ln(GVA per worker). All columns are estimated using a reformulated Mundlak model. Standard errors are clustered at the establishment level. ***, **, and * denote significance at the 1%, 5%, and 10% levels

Source: Authors’ own calculations using ONS ARDx and MES survey

**Table 13:** Robustness checks: alternative models

Dependent variable: ln(GVA per worker)	(1)	(2)	(3)
	RE	OLS	LP
Score	0.989***	1.028***	1.094***
	(0.090)	(0.095)	(0.016)
Score × family ownership	− 0.393***	− 0.445***	− 0.449***
	(0.098)	(0.102)	(0.013)
Family ownership	0.134**	0.168***	0.093***
	(0.062)	(0.065)	(0.031)
Controls	Yes	Yes	Yes
Industry FE	Yes	Yes	Yes
Region FE	Yes	Yes	Yes
Year FE	Yes	Yes	Yes
*N* of observations	16,340	16,339	16,339
*N* of clusters	12,010	12,009	12,009
*R*-sq	0.317	0.318	

The dependent variable is ln(GVA per worker). Columns (1)–(3) report estimates from random effects (RE), ordinary least squares (OLS), and Levinsohn–Petrin (LP) models, respectively. Time-varying control variables include the natural logarithm of the number of employees, the natural logarithm of capital stock per worker, and the natural logarithm of firm age. Time-invariant firm characteristics include company legal status, exporter status, foreign ownership, managers’ university education, and workers’ university education. Industry, region, and year dummies are included in all specifications. Standard errors are clustered at the establishment level and reported in parentheses. ***, **, and * denote statistical significance at the 1%, 5%, and 10% levels, respectively

Source: Authors’ own calculations using ONS ARDx and MES surveys

**Table 14:** Robustness checks: alternative measures of score

Dependent variable: ln(GVA per worker)						
	(1)		(2)		(3)	
	Within	Between	Within	Between	Within	Between
Score-14	0.586***	1.149***				
	(0.203)	(0.102)				
Score-14 × family ownership	− 0.081	− 0.518***				
	(0.234)	(0.110)				
*Z*-score			0.094**	0.213***		
			(0.039)	(0.020)		
*Z*-score × family ownership			0.010	− 0.094***		
			(0.047)	(0.021)		
PCA score					0.046***	0.089***
					(0.018)	(0.009)
PCA score × family ownership					− 0.004	− 0.039***
					(0.021)	(0.009)
Family ownership		0.209***		− 0.077***		− 0.076***
		(0.067)		(0.019)		(0.019)
Controls	Yes		Yes		Yes	
Industry FE	Yes		Yes		Yes	
Region FE	Yes		Yes		Yes	
Year FE	Yes		Yes		Yes	
*N* of observations	16,340		16,340		16,340	
*N* of cluster	12,010		12,010		12,010	
Overall *R*-sq	0.322		0.322		0.322	

The dependent variable is ln(GVA per worker). All columns are estimated using a reformulated Mundlak model. Score-14 is defined as the unweighted average of management practice questions 1–14. *Z*-score is the standardised unweighted average score across all 16 management practice questions. PCA score is the first principal component extracted from all 16 management practice questions. Time-varying control variables include the natural logarithm of the number of employees, the natural logarithm of capital stock per worker, and the natural logarithm of firm age. Time-invariant firm characteristics include company legal status, exporter status, foreign ownership, managers’ education, and workers’ education. Industry, region, and year dummies are included in all specifications. Standard errors are clustered at the establishment level and reported in parentheses. ***, **, and * denote statistical significance at the 1%, 5%, and 10% levels, respectively

Source: Authors’ own calculations using ONS ARDx and MES surveys

**Table 15:** Robustness checks: alternative dependent variables

Dependent variable:	TFP		ln(output per worker)		ln(turnover per worker)	
	(1)		(2)		(3)	
	Within	Between	Within	Between	Within	Between
Score	0.431**	0.881***	0.447***	0.891***	0.023	1.185***
	(0.199)	(0.097)	(0.135)	(0.091)	(0.066)	(0.105)
Score × family ownership	0.014	− 0.335***	− 0.080	− 0.265**	0.085	− 0.316***
	(0.235)	(0.107)	(0.166)	(0.098)	(0.078)	(0.110)
Family ownership		0.150**		0.059		0.094
		(0.068)		(0.061)		(0.069)
Controls	Yes		Yes		Yes	
Industry FE	Yes		Yes		Yes	
Region FE	Yes		Yes		Yes	
Year FE	Yes		Yes		Yes	
*N* of observations	16,340		16,340		31,156	
*N* of cluster	12,010		12,010		17,075	
Overall *R*-sq	0.219		0.549		0.360	

The dependent variables are total factor productivity (TFP) Levinsohn–Petrin (LP) estimator, ln(output per worker), and ln(turnover per worker), respectively. All columns are estimated using a reformulated Mundlak model. Columns (1) and (2) are estimated using the merged ARDx–MES sample, while column (3) uses the MES sample only. ln(turnover per worker) is obtained from the MES; due to the lack of capital stock information in the original MES, capital stock is not controlled for in column (3). In column (2), where ln(output per worker) is used as the dependent variable, ln(intermediate input per worker) is additionally included as a control variable. All columns include time-varying controls and firm-level time-invariant characteristics, as well as industry, region, and year fixed effects. Standard errors are clustered at the establishment level and reported in parentheses. ***, **, and * denote statistical significance at the 1%, 5%, and 10% levels, respectively

Source: Authors’ own calculations using ONS ARDx and MES surveys

**Table 16:** Robustness checks: fully interacted specification

Dependent variable: ln(GVA per worker)		
	(1)	
	Within	Between
Score	0.457***	1.026***
	(0.195)	(0.104)
ln(employees)	− 0.478***	− 0.021*
	(0.085)	(0.011)
ln(capital stock per worker)	0.051	0.164***
	(0.058)	(0.014)
ln(firm age)	0.165**	0.060***
	(0.074)	(0.023)
Family ownership		0.148
		(0.137)
Foreign ownership		0.159***
		(0.031)
Company		0.438***
		(0.048)
Exporter		0.106***
		(0.034)
Manager education		0.118***
		(0.031)
Worker education		0.176***
		(0.040)
Score × family ownership	0.029	− 0.395***
	(0.232)	(0.117)
ln(employees) × family ownership	− 0.023	− 0.012
	(0.144)	(0.015)
ln(capital stock per worker) × family ownership	0.146*	0.012
	(0.079)	(0.015)
ln(firm age) × family ownership	− 0.116	0.034
	(0.109)	(0.030)
Foreign ownership × family ownership		− 0.013
		(0.057)
Company × family ownership		− 0.064
		(0.072)
Exporter × family ownership		0.051
		(0.037)
Manager education × family ownership		− 0.103***
		(0.039)
Worker education × family ownership		− 0.031
		(0.053)
Industry FE	Yes	
Region FE	Yes	
Year FE	Yes	
*N* of observations	16,340	
*N* of cluster	12,010	
Overall *R*-sq	0.314	

The dependent variable is ln(GVA per worker). All columns are estimated using a reformulated Mundlak model. Standard errors are clustered at the establishment level. ***, **, and * denote significance at the 1%, 5%, and 10% levels

Source: Authors’ own calculations using ONS ARDx and MES surveys

**Table 17:** Robustness checks: 2SLS results

Dependent variable: ln(GVA per worker)				
	(1)		(2)	
	IV-mean		IV-lag	
	Within	Between	Within	Between
Score	1.552***	1.838***	2.275**	1.032***
	(0.566)	(0.344)	(0.969)	(0.184)
Score × family ownership	0.195	− 1.201***	− 0.284	− 0.508**
	(0.709)	(0.325)	(1.184)	(0.202)
Family ownership		0.615***		0.197
		(0.192)		(0.131)
Controls	Yes		Yes	
Industry FE	Yes		Yes	
Region FE	Yes		Yes	
Year FE	Yes		Yes	
*N* of observations	16,340		4442	
*N* of groups	12,010		4442	
*R*-sq	0.314		0.315	

The dependent variable is ln(GVA per worker). Columns (1)–(2) report 2SLS random effects estimates based on a reformulated Mundlak model. In column (1), management practices (within effect and between effect) are instrumented using the average management score of firms operating in the same two-digit industry, region, and survey wave; the interaction term is instrumented by interacting these averages with family ownership. Column (2) uses the one-period lag of within-firm management practices as an alternative instrument. Time-varying controls include the natural logarithm of the number of employees, capital stock per worker, and firm age. Time-invariant firm characteristics include company legal status, exporter status, foreign ownership, managers’ education, and workers’ education. Industry, region, and year fixed effects are included in all specifications. Standard errors are clustered at the establishment level and reported in parentheses. ***, **, and * denote statistical significance at the 1%, 5%, and 10% levels, respectively

Source: Authors’ own calculations using ONS ARDx and MES surveys

**Table 18:** Heterogeneity analysis: knowledge intensive and low-skilled services

Dependent variable: ln(GVA per worker)				
	(1)		(2)	
	Knowledge intensive		Low-skilled	
	Within	Between	Within	Between
Score	0.833**	1.411***	0.053	1.130***
	(0.333)	(0.204)	(0.402)	(0.161)
Score × family ownership	− 0.373	− 0.673***	0.669	− 0.452***
	(0.437)	(0.229)	(0.447)	(0.173)
Family ownership		0.323**		0.155
		(0.145)		(0.109)
Controls	Yes		Yes	
Industry FE	Yes		Yes	
Region FE	Yes		Yes	
Year FE	Yes		Yes	
*N* of observations	4646		5914	
*N* of clusters	3575		4397	
Overall *R*-sq	0.361		0.313	

The dependent variable is ln(GVA per worker). Both columns are estimated using a reformulated Mundlak model. Manufacturing sectors are defined as industries with 2-digit SIC codes 10–33, while service sectors correspond to SIC codes 45–96. Knowledge-intensive services include industries with 2-digit SIC codes 50, 51, 58–66, 69–75, 78, 80, 84–88, and 90–93. All remaining service industries are classified as low-skilled services. Time-varying controls include the natural logarithm of the number of employees, capital stock per worker, and firm age. Time-invariant firm characteristics include company legal status, exporter status, foreign ownership, managers’ education, and workers’ education. Industry, region, and year fixed effects are included in all specifications. Standard errors are clustered at the establishment level and reported in parentheses. ***, **, and * denote statistical significance at the 1%, 5%, and 10% levels, respectively

Source: Authors’ own calculations using ONS ARDx and MES surveys

**Table 19:** Heterogeneity analysis: further details by size and sectors

Dependent variable: ln(GVA per worker)										
	(1)		(2)		(3)		(4)		(5)	
	SMEs services		SMEs manufacturing		Large services		SMEs knowledge intensive services		SMEs low-skilled services	
	Within	Between	Within	Between	Within	Between	Within	Between	Within	Between
Management score	0.768*	1.360***	0.142	0.539***	0.203	0.880***	1.513***	1.259***	0.210***	1.279***
	(0.355)	(0.156)	(0.284)	(0.156)	(0.344)	(0.245)	(0.458)	(0.131)	(0.521)	(0.182)
Management score × family ownership	− 0.070	− 0.663***	0.058	− 0.198	0.186	− 0.402	− 0.956*	− 0.661**	0.544	− 0.664**
	(0.399)	(0.166)	(0.380)	(0.178)	(0.493)	(0.330)	(0.558)	(0.282)	(0.564)	(0.195)
Family ownership		0.292***		0.044		0.160		0.345**		0.225**
		(0.101)		(0.111)		(0.233)		(0.090)		(0.118)
Controls	Yes		Yes		Yes		Yes		Yes	
Industry FE	Yes		Yes		Yes		Yes		Yes	
Region FE	Yes		Yes		Yes		Yes		Yes	
Year FE	Yes		Yes		Yes		Yes		Yes	
*N* of observations	7974		3665		2586		3246		4728	
*N* of clusters	6216		2498		1834		2615		3605	
Overall *R*-sq	0.326		0.157		0.389		0.369		0.299	

The dependent variable is the natural logarithm of GVA per worker. All columns are estimated using a reformulated Mundlak model. Columns (1)–(5) report estimates for subsamples of SMEs services sector, SMEs manufacturing, large services sector, SMEs knowledge intensive, and SMEs low skilled industries, respectively. All specifications are estimated using a reformulated Mundlak model. Time varying control variables include the natural logarithm of the number of employees, the natural logarithm of capital stock per worker, and the natural logarithm of firm age. Time invariant firm characteristics include company legal status, exporter status, foreign ownership, manager university educated, and worker university educated. Industry, region, and year fixed effects are included in all specifications. Standard errors are clustered at the establishment level and reported in parentheses. *, **, and *** denote statistical significance at the 10%, 5%, and 1% levels, respectively

Source: Authors’ own calculations using ONS ARDx and MES surveys

**Table 20:** Linear relationship: alternative models

Dependent variable: ln(GVA per worker)			
	(1)	(2)	(3)
	RE	OLS	LP
Score	0.707***	0.704***	1.407***
	(0.046)	(0.047)	(0.331)
Control	Yes	Yes	Yes
Industry FE	Yes	Yes	Yes
Region FE	Yes	Yes	Yes
Year FE	Yes	Yes	Yes
*N* of observations	16,340	16,339	16,339
*N* of cluster	12,010	12,009	12,009
Overall *R*-sq	0.316	0.317	-

The dependent variable is ln(GVA per worker). Columns (1)–(3) report estimates from random effects (RE), ordinary least squares (OLS), and Levinsohn–Petrin (LP) specifications, respectively. Time-varying control variables include the natural logarithm of the number of employees, the natural logarithm of capital stock per worker, and the natural logarithm of firm age. Time-invariant firm characteristics include company legal status, exporter status, foreign ownership, managers’ university education, and workers’ university education. Industry, region, and year fixed effects are included in all specifications. Standard errors are clustered at the establishment level and reported in parentheses. ***, **, and * denote statistical significance at the 1%, 5%, and 10% levels, respectively

Source: Authors’ own calculations using ONS ARDx and MES surveys

**Table 21:** Linear relationship: alternative measures of management practices score

Dependent variable: ln(GVA per worker)						
	(1)		(2)		(3)	
	Within	Between	Within	Between	Within	Between
Score-14	0.526***	0.773***				
	(0.104)	(0.051)				
*Z*-score			0.099***	0.145***		
			(0.022)	(0.010)		
PCA score					0.043***	0.060***
					(0.009)	(0.004)
Controls	Yes		Yes		Yes	
Industry FE	Yes		Yes		Yes	
Region FE	Yes		Yes		Yes	
Year FE	Yes		Yes		Yes	
*N* of observations	16,340		16,340		16,340	
*N* of cluster	12,010		12,010		12,010	
Overall *R*-sq	0.321		0.320		0.319	

The dependent variable is ln(GVA per worker). All specifications are estimated using a reformulated Mundlak model. Score-14 is defined as the unweighted average of management practice questions 1–14. *Z*-score is the standardised unweighted average score across all 16 management practice questions. PCA score is the first principal component extracted from all 16 management practice questions. Time-varying control variables include the natural logarithm of the number of employees, the natural logarithm of capital stock per worker, and the natural logarithm of firm age. Time-invariant firm characteristics include company legal status, exporter status, foreign ownership, managers’ education, and workers’ education. Industry, region, and year fixed effects are included in all specifications. Standard errors are clustered at the establishment level and reported in parentheses. ***, **, and * denote statistical significance at the 1%, 5%, and 10% levels, respectively

Source: Authors’ own calculations using ONS ARDx and MES surveys

**Table 22:** Linear relationship: alternative dependent variables

Dependent variable:	TFP		ln(output per worker)		ln(turnover per worker)	
	(1)		(2)		(3)	
	Within	Between	Within	Between	Within	Between
Score	0.437***	0.644***	0.459***	0.770***	0.079**	0.958***
	(0.109)	(0.046)	(0.091)	(0.049)	(0.036)	(0.053)
Controls	Yes		Yes		Yes	
Industry FE	Yes		Yes		Yes	
Region FE	Yes		Yes		Yes	
Year FE	Yes		Yes		Yes	
*N* of observations	16,340		16,340		31,156	
*N* of cluster	12,010		12,010		17,075	
Overall *R*-sq	0.218		0.511		0.360	

The dependent variables are total factor productivity (TFP) Levinsohn–Petrin (LP) estimator, ln(output per worker), and ln(turnover per worker), respectively. Columns (1) and (2) are estimated using the merged ARDx–MES sample, while column (3) uses the MES sample only. ln(turnover per worker) is obtained from the MES; due to the lack of capital stock information in the original MES, capital stock is not controlled for in column (3). In column (2), where ln(output per worker) is used as the dependent variable, ln(intermediate input per worker) is additionally included as a control variable. All specifications include time-varying controls and firm-level time-invariant characteristics, as well as industry, region, and year fixed effects. Standard errors are clustered at the establishment level and reported in parentheses. ***, **, and * denote statistical significance at the 1%, 5%, and 10% levels, respectively

Source: Authors’ own calculations using ONS ARDx and MES surveys

**Table 23:** Linear relationship: 2SLS results

Dependent variable: ln(GVA per worker)				
	(1)		(2)	
	IV-mean		IV-lag	
	Within	Between	Within	Between
Score	1.685***	1.004***	2.045***	0.653***
	(0.394)	(0.216)	(0.579)	(0.092)
Control	Yes		Yes	
Industry FE	Yes		Yes	
Region FE	Yes		Yes	
Year FE	Yes		Yes	
*N* of observations	16,340		4442	
*N* of groups	12,010		4442	
*R*-sq	0.315		0.314	

The dependent variable is ln(GVA per worker). Columns (1)–(2) report 2SLS random effects estimates based on a reformulated Mundlak specification. In column (1), management practices (within effect and between effect) are instrumented using the average management score of firms operating in the same two-digit industry, region, and survey wave; the interaction term is instrumented by interacting these averages with family ownership. Column (2) uses the one-period lag of within-firm management practices as an alternative instrument. Time-varying controls include the natural logarithm of the number of employees, capital stock per worker, and firm age. Time-invariant firm characteristics include company legal status, exporter status, foreign ownership, managers’ education, and workers’ education. Industry, region, and year fixed effects are included in all specifications. Standard errors are clustered at the establishment level and reported in parentheses. ***, **, and * denote statistical significance at the 1%, 5%, and 10% levels, respectively

Source: Authors’ own calculations using ONS ARDx and MES surveys

**Table 24:** Linear relationship: firm size and sector heterogeneity

Dependent variable: ln(GVA per worker)												
	(1)		(2)		(3)		(4)		(5)		(6)	
	SMEs		Large		Manufacturing		Services		Knowledge intensive services		Low-skilled services	
	Within	Between	Within	Between	Within	Between	Within	Between	Within	Between	Within	Between
Score	0.537***	0.720***	0.376*	0.757***	0.262	0.421***	0.571***	0.855***	0.629***	1.011***	0.524***	0.779***
	(0.125)	(0.052)	(0.214)	(0.168)	(0.181)	(0.081)	(0.143)	(0.066)	(0.219)	(0.118)	(0.190)	(0.078)
Control	Yes		Yes		Yes		Yes		Yes		Yes	
Industry FE	Yes		Yes		Yes		Yes		Yes		Yes	
Region FE	Yes		Yes		Yes		Yes		Yes		Yes	
Year FE	Yes		Yes		Yes		Yes		Yes		Yes	
*N* of observations	13,032		3308		4214		10,560		4646		5914	
*N* of cluster	9806		2323		2849		7965		3575		4397	
Overall *R*-sq	0.302		0.413		0.170		0.330		0.359		0.311	

The dependent variable is ln(GVA per worker). SMEs are defined as firms with fewer than 250 employees, while large firms have 250 employees or more. Manufacturing sectors are defined as industries with 2-digit SIC codes 10–33, while service sectors correspond to 2-digit SIC codes 45–96. Knowledge-intensive services include industries with 2-digit SIC codes 50, 51, 58–66, 69–75, 78, 80, and 84–88, as well as 90–93. All remaining service industries are classified as low-skilled services. Time-varying controls include the natural logarithm of the number of employees, capital stock per worker, and firm age. Time-invariant firm characteristics include company legal status, exporter status, foreign ownership, managers’ education, and workers’ education. Industry, region, and year fixed effects are included in all specifications. Standard errors are clustered at the establishment level and reported in parentheses. ***, **, and * denote statistical significance at the 1%, 5%, and 10% levels, respectively

Source: Authors’ own calculations using ONS ARDx and MES surveys

**Table 25:** Linear relationship: sub-score analysis

Dependent variable: ln(GVA per worker)												
	(1)		(2)		(3)		(4)		(5)		(6)	
	Within	Between	Within	Between	Within	Between	Within	Between	Within	Between	Within	Between
Monitoring	0.238**	0.449***										
	(0.093)	(0.048)										
Targets			0.146***	0.279***								
			(0.054)	(0.032)								
Incentives					0.212***	0.501***						
					(0.059)	(0.033)						
Training							0.241***	0.295***				
							(0.064)	(0.034)				
Managers’ score									0.362***	0.642***		
									(0.082)	(0.045)		
Workers’ score											0.406***	0.639***
											(0.091)	(0.043)
Control	Yes		Yes		Yes		Yes		Yes		Yes	
Industry FE	Yes		Yes		Yes		Yes		Yes		Yes	
Region FE	Yes		Yes		Yes		Yes		Yes		Yes	
Year FE	Yes		Yes		Yes		Yes		Yes		Yes	
*N* of observations	16,340		16,340		16,340		16,340		16,340		16,340	
*N* of cluster	12,010		12,010		12,010		12,010		12,010		12,010	
Overall *R*-sq	0.312		0.312		0.320		0.312		0.318		0.319	

The dependent variable is ln(GVA per worker). All specifications are estimated using a reformulated Mundlak model. All remaining service industries are classified as low-skilled services. Columns (1)–(6) report results for different dimensions of management practices. Monitoring is constructed as the average score of survey questions Q1–4, targets as the average score of Q5–8, incentives as the average score of Q9–12, and training as the average score of Q13–14. Managers’ score is constructed as the average score of survey questions Q3, 7, 9, 11, 13, while workers’ score is constructed as the average score of Q4, 8, 10, 12, 14. Time varying control variables include the natural logarithm of the number of employees, the natural logarithm of capital stock per worker, and the natural logarithm of firm age. Time invariant firm characteristics include company legal status, exporter status, foreign ownership, manager university educated, and worker university educated. Industry, region, and year fixed effects are included in all specifications. Standard errors are clustered at the establishment level and reported in parentheses. *, **, and *** denote statistical significance at the 10%, 5%, and 1% levels, respectively

Source: Authors’ own calculations using ONS ARDx and MES surveys

**Table 26:** MES questionnaire items and scores

No	Question	Response	Score
***Monitoring***			
1	In general, what was the most common response to problems faced within your business?	We resolved the problems but did not take further action	1/3
		We resolved the problems and took action to try to ensure they did not happen again	2/3
		We resolved the problems and had a continuous improvement process to anticipate similar problems in advance	1
		No action was taken	0
2	How many key performance indicators (KPIs) did you monitor?	1–2 key performance indicators	1/3
		3–9 key performance indicators	2/3
		10 or more key performance indicators	1
		No key performance indicators	0
3	How frequently was progress against the key performance indicators (KPIs) reviewed by managers?	Annually	1/6
		Quarterly	2/6
		Monthly	1/2
		Weekly	2/3
		Daily	5/6
		Hourly or more frequently	1
		Never	0
4	How frequently was progress against the key performance indicators (KPIs) reviewed by non-managers?	Yearly	1/6
		Quarterly	2/6
		Monthly	1/2
		Weekly	2/3
		Daily	5/6
		Hourly or more frequently	1
		Never	0
***Targets***			
5	Which of the following best describes the main timeframes for achieving targets within your business?	Main time frame was less than one year	1/3
		Main time frame was one year or more	2/3
		Combination of time frames of less than and more than one year	1
		There were no targets	0
6	How easy or difficult was it to achieve these targets?	Very easy—Possible to achieve without much effort	0
		Quite easy—Possible to achieve with some effort	1/2
		Neither easy nor difficult—Possible to achieve with normal effort	3/4
		Quite difficult—Possible to achieve with more than normal effort	1
		Very difficult—Possible to achieve with extraordinary effort	1/4
7	Approximately what proportion of managers were aware of these targets?	All	1
		Most	2/3
		Some	1/3
		None	0
8	Approximately what proportion of non-managers were aware of these targets?	All	1
		Most	2/3
		Some	1/3
		None	0
***Incentives***			
9	What were performance bonuses for managers usually based on within your business?	Their own performance as measured by targets	1
		Their team’s or shift’s performance as measured by targets	4/5
		Their site’s performance as measured by targets	3/5
		The business’ performance as measured by targets	2/5
		Performance bonuses were not related to targets	1/5
		No performance bonuses	0
10	What were performance bonuses for non-managers usually based on within your business?	Their own performance as measured by targets	1
		Their team’s or shift’s performance as measured by targets	4/5
		Their site’s performance as measured by targets	3/5
		The business’ performance as measured by targets	2/5
		Performance bonuses were not related to targets	1/5
		No performance bonuses	0
11	How were managers usually promoted within your business?	Based solely on performance or ability	1
		Based partly on performance or ability, and partly on other factors	2/3
		Based mainly on factors other than performance or ability	1/3
		No managers were promoted	0
12	How were non-managers usually promoted within your business?	Based solely on performance or ability	1
		Based partly on performance or ability, and partly on other factors	2/3
		Based mainly on factors other than performance or ability	1/3
		No managers were promoted	0
***Training***			
13	On average how many days training and development did managers undertake within your business?	Less than a day	0
		1 day	1/4
		2–4 days	1/2
		5–10 days	3/4
		More than 10 days	1
14	On average how many days training and development did non-managers undertake within your business?	Less than a day	0
		1 day	1/4
		2–4 days	1/2
		5–10 days	3/4
		More than 10 days	1
***Other***			
15	Which of the following best describes the timeframe that action was taken to address under-performance among managers within your business?	Within 6 months of identifying under-performance	1
		After 6 months of identifying under-performance	1/2
		No action was taken to address under-performance	0
		There was no under-performance	0
16	Which of the following best describes the timeframe that action was taken to address under-performance among non-managers within your business?	Within 6 months of identifying under-performance	1
		After 6 months of identifying under-performance	1/2
		No action was taken to address under-performance	0
		There was no under-performance	0

Source: ONS Management and Expectations Survey

## Footnotes

1 Throughout the paper, the terms *firm* and *establishment* are used interchangeably, as the analysis is conducted at the establishment level.

2 Academic research using UK MES data remains relatively limited. Existing studies include Bloom et al. (2021), which analyse the 2016 MES cross-sectional data, as well as some preliminary descriptive analyses published by the UK Office for National Statistics (ONS) (see, for example https://www.ons.gov.uk/economy/economicoutputandproductivity/productivitymeasures/bulletins/managementpracticesintheuk/2016to2023). Existing studies for the USA and Germany focus primarily on manufacturing sector (Bloom & Van Reenen, 2007; Bloom et al., 2019, Broszeit et al., 2019, Jirjahn et al., 2024).

3 For example, the Management and Organizational Practices Survey (MOPS) for the USA is accessible at https://www.census.gov/data/tables/2015/econ/mops/2015-survey-release.html. Twelve out of sixteen questions are identical in the MOPS and the MES.

4 ARDx is a research database created by the ONS through the harmonisation and linking of data from the Annual Business Inquiry (1998–2008) and the Annual Business Survey (2009 onwards). It also incorporates employment information from the Business Register and Employment Survey. The database provides official estimates of capital stock based on the perpetual inventory method, which we use to estimate productivity. The ABS is a standalone annual survey that primarily provides cross-sectional financial data and is not well suited to longitudinal analysis.

5 We performed data cleaning such as dropping (1) observations with missing values in essential variables, (2) a few observations with inconsistent family ownership information (nonfamily-owned firms but run by family members and family-owned firms but not reporting management structure), (3) firms that change family ownership status over the 3-year panel (272 firms). The last step reduces possible endogeneity between changes in family ownership status and productivity. Table 5 in the Appendix shows our data cleaning and sample construction process.

6 Questions 15 and 16 are excluded from the sub-score analysis because their response categories are not comparable with those used in the US and German surveys. In particular, both the absence of underperformance and cases in which no action is taken to address underperformance are assigned a score of zero, potentially obscuring meaningful differences in management practices.

7 Table 12 in the Appendix contains all estimated coefficients. The production function is correctly specified as it exhibits the diminishing returns to production inputs. Firm age, foreign ownership, export status, being a limited company, and higher education of managers and employees are positively associated with the labour productivity, as expected. Family ownership, in turn, is negatively associated with productivity.

8 To assess the robustness of the linear relationship between management practices and productivity, for completeness, we implement a series of checks that are consistent with those performed for the regressions with the moderating effect of family ownership, presented in Sections 4.2, 4.3, and 4.4. These checks include alternative model specifications, alternative dependent and independent variables, as well as analyses based on management practices sub-scores, and heterogeneity by firm size and sector. The results are reported in Appendix Tables 20, 21, 22, 23, 24, and 25.

9 Comparing our results with evidence from the USA and Germany for the manufacturing sector, Table 24 shows that the average productivity effect of management practices in UK manufacturing is relatively smaller. Moreover, we find that the productivity returns to management practices are lower in manufacturing than in the service sector, which contrasts with the evidence from Mexico reported in Bloom et al. (2022).

10 4.8%=100% × (exp(0.472 × 0.1) − 1) and 7.6% = 100% × (exp(0.728 × 0.1) − 1)). Note that betweenfirm effect may be upward biased if management practices are endogenous to productivity, while within-firm effect is less susceptible to reverse causality. Given that, the true long-run effect of managerial practices is most likely between 4.8 and 7.6%.

11 Wald ${\chi }^{2}$ test, ${\chi }^{2}=0.23$, *p*-value = 0.631.

12 Firm-level TFP is obtained as the residual from the estimated production functions on the ARDx data for 2016–2020 at the two-digit SIC level using the Levinsohn–Petrin (LP) method, with labour and capital as inputs. Then it is merged with the MES data, and used in the Mundlak model. This is in contrast to the results in column (3) of Table 13 where the LP estimator is applied directly to the merged ARDx–MES sample to relate gross value added to management practices and their interaction with family ownership.

13 We follow Eurostat definition of knowledge-intensive services (https://ec.europa.eu/eurostat/statisticsexplained/index.php?title=Glossary:Knowledge-intensive_services_(KIS)).

## References

[CR1] Office for National Statistics. (2023). *Annual Respondents Database X, 1997–2020: Secure Access*. 10.5255/UKDA-SN-7989-5

[CR2] Office for National Statistics. (2025). *Management and Expectations Survey, 2016–2023: Secure Access*. 10.5255/UKDA-SN-8557-6

[CR3] Aghion, P., & Holden, R. (2011). Incomplete contracts and the theory of the firm: What have we learned over the past 25 years? *Journal of Economic Perspectives,**25*(2), 181–197. 10.1257/jep.25.2.181

[CR4] Aguilera, R. V., Crespi-Cladera, R., Martín-Oliver, A., & Pascual-Fuster, B. (2025). Ownership, control, and productivity: Family firms in comparative perspective. *Journal of Management,**51*(8), 3321–3351. 10.1177/01492063241259964

[CR5] Alonso, J. M., & Andrews, R. (2016). How privatization affects public service quality: An empirical analysis of prisons in England and Wales, 1998–2012. *International Public Management Journal,**19*(2), 235–263. 10.1080/10967494.2015.1048913

[CR6] Aragón, M. I. B., Jiménez, D. J., & Valle, R. S. (2014). Training and performance: The mediating role of organizational learning. *BRQ Business Research Quarterly,**17*(3), 161–173. 10.1016/j.cede.2013.05.003

[CR7] Attig, N., & Cleary, S. (2014). Organizational capital and investment-cash flow sensitivity: The effect of management quality practices. *Financial Management,**43*(3), 473–504. 10.1111/fima.12046

[CR8] Bell, A., Fairbrother, M., & Jones, K. (2019). Fixed and random effects models: Making an informed choice. *Quality & Quantity,**53*(2), 1051–1074. 10.1007/s11135-018-0802-x

[CR9] Bellisario, A., Pavlov, A., & van der Steen, M. P. (2021). The role of performance measurement in aligning operations with strategy: Sustaining cognitive processes of internal alignment. *International Journal of Operations & Production Management,**41*(12), 1879–1907. 10.1108/ijopm-02-2021-0081

[CR10] Bender, S., Bloom, N., Card, D., Van Reenen, J., & Wolter, S. (2018). Management practices, workforce selection, and productivity. *Journal of Labor Economics,**36*(S1), S371–S409. 10.1086/694107

[CR11] Bennedsen, M., Gonzalez, F. P., & Wolfenzon, D. (2010). The governance of family firms. In *Corporate Governance* (pp. 371–389). 10.1002/9781118258439.ch19

[CR12] Blader, S., Gartenberg, C., & Prat, A. (2019). The contingent effect of management practices. *The Review of Economic Studies,**87*(2), 721–749. 10.1093/restud/rdz034

[CR13] Bloom, N., Brynjolfsson, E., Foster, L., Jarmin, R., Patnaik, M., Saporta-Eksten, I., & Van Reenen, J. (2019). What drives differences in management practices? *American Economic Review,**109*(5), 1648–1683. 10.1257/aer.20170491

[CR14] Bloom, N., Brynjolfsson, E., Foster, L., Jarmin, R. S., Saporta-Eksten, I., & Van Reenen, J. (2013). Management in America. *US Census Bureau Center for Economic Studies*, *CES-WP-13–01*. 10.2139/ssrn.2200954

[CR15] Bloom, N., Eifert, B., Mahajan, A., McKenzie, D., & Roberts, J. (2012a). Does management matter? Evidence from India. *The Quarterly Journal of Economics,**128*(1), 1–51. 10.1093/qje/qjs044

[CR16] Bloom, N., Genakos, C., Sadun, R., & Van Reenen, J. (2012b). Management practices across firms and countries. *Academy of Management Perspectives,**26*(1), 12–33. 10.5465/amp.2011.0077

[CR17] Bloom, N., Iacovone, L., Pereira-Lopez, M., & Van Reenen, J. (2022). Management and misallocation in Mexico. *National Bureau of Economic Research Working Paper Series*, *No. 29717*. 10.3386/w29717

[CR18] Bloom, N., Sadun, R., & Van Reenen, J. (2016). Management as a technology? *National Bureau of Economic Research Working Paper Series*, *No. 22327*. 10.3386/w22327

[CR19] Bloom, N., & Van Reenen, J. (2007). Measuring and explaining management practices across firms and countries. *The Quarterly Journal of Economics,**122*(4), 1351–1408. 10.1162/qjec.2007.122.4.1351

[CR20] Bradbury, J. C. (2019). Monitoring and employee shirking: Evidence from MLB umpires. *Journal of Sports Economics,**20*(6), 850–872. 10.1177/1527002518808350

[CR21] Broszeit, S., Laible, M.-C., Fritsch, U., & Görg, H. (2019). Management practices and productivity in Germany. *German Economic Review,**20*(4), e657–e705. 10.1111/geer.12187

[CR22] Brynjolfsson, E., Hitt, L. M., & Yang, S. (2002). Intangible assets: Computers and organizational capital. *Brookings Papers on Economic Activity,**2002*(1), 137–181.

[CR23] Cette, G., Lopez, J., Mairesse, J., & Nicoletti, G. (2024). Trust, intangible assets, and productivity. *National Bureau of Economic Research Working Paper Series*, *No. 32513*. 10.3386/w32513

[CR24] Chrisman, J. J., Devaraj, S., & Patel, P. C. (2017). The impact of incentive compensation on labor productivity in family and nonfamily firms. *Family Business Review,**30*(2), 119–136. 10.1177/0894486517690052

[CR25] Chrisman, J. J., Fang, H. C., & Skorodziyevskiy, V. (2025). Toward a property rights theory of the family firm. *Journal of Management,**0*(0), Article 01492063251355258. 10.1177/01492063251355258

[CR26] Christ, M. H. (2013). An experimental investigation of the interactions among intentions, reciprocity, and control. *Journal of Management Accounting Research,**25*(1), 169–197. 10.2308/jmar-50443

[CR27] Cornett, M. M., Marcus, A. J., Saunders, A., & Tehranian, H. (2007). The impact of institutional ownership on corporate operating performance. *Journal of Banking & Finance,**31*(6), 1771–1794. 10.1016/j.jbankfin.2006.08.006

[CR28] Daspit, J. J., Chrisman, J. J., Ashton, T., & Evangelopoulos, N. (2021). Family firm heterogeneity: A definition, common themes, scholarly progress, and directions forward. *Family Business Review,**34*(3), 296–322. 10.1177/08944865211008350

[CR29] Davis, J. H., Schoorman, F. D., & Donaldson, L. (1997). Toward a stewardship theory of management. *Academy of Management Review,**22*(1), 20–47. 10.5465/amr.1997.9707180258

[CR30] De Massis, A., Frattini, F., Majocchi, A., & Piscitello, L. (2018). Family firms in the global economy: Toward a deeper understanding of internationalization determinants, processes, and outcomes. *Global Strategy Journal,**8*(1), 3–21. 10.1002/gsj.1199

[CR31] Diaz-Moriana, V., Clinton, E., & Kammerlander, N. (2024). Untangling goal tensions in family firms: A sensemaking approach. *Journal of Management Studies,**61*(1), 69–109. 10.1111/joms.12845

[CR32] Fang, H. C., Randolph, R. V. D. G., Memili, E., & Chrisman, J. J. (2016). Does size matter? The moderating effects of firm size on the employment of nonfamily managers in privately held family SMEs. *Entrepreneurship Theory and Practice,**40*(5), 1017–1039. 10.1111/etap.12156

[CR33] Favero, N., Meier, K. J., & O’Toole, L. J., Jr. (2014). Goals, trust, participation, and feedback: Linking internal management with performance outcomes. *Journal of Public Administration Research and Theory,**26*(2), 327–343. 10.1093/jopart/muu044

[CR34] Fisman, R., & Svensson, J. (2007). Are corruption and taxation really harmful to growth? Firm level evidence. *Journal of Development Economics,**83*(1), 63–75. 10.1016/j.jdeveco.2005.09.009

[CR35] Fiss, P. C. (2008). Institutions and corporate governance. *The Sage handbook of organizational institutionalism*, 389–410.

[CR36] Gosnell, G. K., List, J. A., & Metcalfe, R. D. (2020). The impact of management practices on employee productivity: A field experiment with airline captains. *Journal of Political Economy,**128*(4), 1195–1233. 10.1086/705375

[CR37] Hart, O., & Moore, J. (2007). Incomplete contracts and ownership: Some new thoughts. *American Economic Review,**97*(2), 182–186. 10.1257/aer.97.2.182

[CR38] Helsen, Z., Lybaert, N., Steijvers, T., Orens, R., & Dekker, J. (2017). Management control systems in family firms: A review of the literature and directions for the future. *Journal of Economic Surveys,**31*(2), 410–435. 10.1111/joes.12154

[CR39] Hoffer Gittell, J. (2002). Coordinating mechanisms in care provider groups: Relational coordination as a mediator and input uncertainty as a moderator of performance effects. *Management Science,**48*(11), 1408–1426. 10.1287/mnsc.48.11.1408.268

[CR40] Ichniowski, C., & Shaw, K. (2003). Beyond incentive pay: Insiders’ estimates of the value of complementary human resource management practices. *Journal of Economic Perspectives,**17*(1), 155–180. 10.1257/089533003321164994

[CR41] Innes, P., & Wiesner, R. (2012). Beyond HRM intensity: Exploring intra-function HRM clusters in SMEs. *Small Enterprise Research,**19*(1), 32–51. 10.5172/ser.2012.19.1.32

[CR42] James, H. S. (1999). Owner as manager, extended horizons and the family firm. *International Journal of the Economics of Business,**6*(1), 41–55. 10.1080/13571519984304

[CR43] Jensen, M. C., & Meckling, W. H. (1979). Theory of the firm: Managerial behavior, agency costs, and ownership structure. In K. Brunner (Ed.), *Economics social institutions: Insights from the conferences on analysis & ideology* (pp. 163–231). Springer Netherlands. 10.1007/978-94-009-9257-3_8

[CR44] Jirjahn, U., Laible, M.-C., & Mohrenweiser, J. (2024). Management practices and productivity: Does employee representation play a moderating role? *Human Resource Management Journal,**34*(1), 236–254. 10.1111/1748-8583.12526

[CR45] Koch, M. J., & McGrath, R. G. (1996). Improving labor productivity: Human resource management policies do matter. *Strategic Management Journal,**17*(5), 335–354. 10.1002/(SICI)1097-0266(199605)17:5<335::AID-SMJ814>3.0.CO;2-R

[CR46] Kotlar, J., & De Massis, A. (2013). Goal setting in family firms: Goal diversity, social interactions, and collective commitment to family–centered goals. *Entrepreneurship Theory and Practice,**37*(6), 1263–1288. 10.1111/etap.12065

[CR47] Kussudyarsana, K., Soepatini, S., Maimun, M. H., & Vemuri, R. (2019). Examining formal and relational governance in family small medium enterprises: Evidence from Indonesia. *Journal of Entrepreneurship in Emerging Economies,**12*(2), 231–257. 10.1108/jeee-10-2018-0108

[CR48] Levinsohn, J., & Petrin, A. (2003). Estimating production functions using inputs to control for unobservables. *The review of economic studies,**70*(2), 317–341. 10.1111/1467-937X.00246

[CR49] Lubatkin, M. H., Ling, Y., & Schulze, W. S. (2007). An organizational justice-based view of self-control and agency costs in family firms. *Journal of Management Studies,**44*(6), 955–971. 10.1111/j.1467-6486.2006.00673.x

[CR50] Martinez Ferrero, J., Rodríguez-Ariza, L., & Bermejo-Sánchez, M. (2016). Is family ownership of a firm associated with the control of managerial discretion and corporate decisions? *Journal of Family Business Management*,* 6*(1). 10.1108/jfbm-06-2015-0022

[CR51] Millar, C. C. J. M., & Choi, C. J. (2011). The innovative future of service industries: (Anti-)globalization and commensuration. *The Service Industries Journal,**31*(1), 21–38. 10.1080/02642069.2010.486030

[CR52] Mundlak, Y. (1978). On the pooling of time series and cross section data. *Econometrica,**46*(1), 69–85. 10.2307/1913646

[CR53] Mustakallio, M., Autio, E., & Zahra, S. A. (2002). Relational and contractual governance in family firms: Effects on strategic decision making. *Family Business Review,**15*(3), 205–222. 10.1111/j.1741-6248.2002.00205.x

[CR54] Owusu-Acheampong, E., Ohene Afriyie, E., Abakah, M. B., Enyan, J. A., & Addaquay, A. S. (2025). A scoping review of human resource management practices in family business. *Journal of Family Business Management*, 1–22. 10.1108/jfbm-03-2025-0066

[CR55] Paşaoğlu, D. (2015). Analysis of the relationship between human resources management practices and organizational commitment from a strategic perspective: Findings from the banking industry. *Procedia - Social and Behavioral Sciences,**207*, 315–324. 10.1016/j.sbspro.2015.10.101

[CR56] Prado, M. (2011). Government policy in the formal and informal sectors. *European Economic Review,**55*(8), 1120–1136. 10.1016/j.euroecorev.2011.04.010

[CR57] Rao, R. V. (2013). Multiple attribute decision making in the manufacturing environment. In *Decision making in manufacturing environment using graph theory and fuzzy multiple attribute decision making methods: Volume 2* (pp. 1–5). Springer London. 10.1007/978-1-4471-4375-8_1

[CR58] Schnieder, C., Schedlinsky, I., Sommer, F., & Wöhrmann, A. (2025). The effects of relative performance information and transparency on knowledge sharing and productive effort. *European Accounting Review,**34*(3), 1195–1220. 10.1080/09638180.2024.2347638

[CR59] Scholes, L., & Wilson, N. (2014). The importance of family firm trusts in family firm governance. *Entrepreneurship Theory and Practice,**38*(6), 1285–1293. 10.1111/etap.12124

[CR60] Scur, D., Ohlmacher, S., Van Reenen, J., Bennedsen, M., Bloom, N., Choudhary, A., Foster, L., Groenewegen, J., Grover, A., Hardeman, S., Iacovone, L., Kambayashi, R., Laible, M.-C., Lemos, R., Li, H., Linarello, A., Maliranta, M., Medvedev, D., Meng, C., … Zimmermann, F. (2024). The international empirics of management. *Proceedings of the National Academy of Sciences,**121*(45), Article e2412205121. 10.1073/pnas.2412205121 10.1073/pnas.2412205121 PMC11551412 39485796

[CR61] Steier, L. (2001). Family firms, plural forms of governance, and the evolving role of trust. *Family Business Review,**14*(4), 353–368. 10.1111/j.1741-6248.2001.00353.x

[CR62] Stewart, A., & Hitt, M. A. (2012). Why can’t a family business be more like a nonfamily business?: Modes of professionalization in family firms. *Family Business Review,**25*(1), 58–86. 10.1177/0894486511421665

[CR63] Verbeeten, F. H. M. (2008). Performance management practices in public sector organizations: Impact on performance. *Accounting, Auditing & Accountability Journal,**21*(3), 427–454. 10.1108/09513570810863996

[CR64] Wooldridge, J. M. (2010). *Econometric analysis of cross section and panel data*. MIT press.

[CR65] Zellweger, T. M., Chrisman, J. J., Chua, J. H., & Steier, L. P. (2019). Social structures, social relationships, and family firms. *Entrepreneurship Theory and Practice,**43*(2), 207–223. 10.1177/1042258718792290

